# Identification of Phytochemicals in Bioactive Extracts of *Acacia saligna* Growing in Australia

**DOI:** 10.3390/molecules28031028

**Published:** 2023-01-19

**Authors:** Anjar P. Asmara, Anchalee Prasansuklab, Tewin Tencomnao, Alison T. Ung

**Affiliations:** 1 School of Mathematical and Physical Sciences, Faculty of Science, University of Technology Sydney, Ultimo, NSW 2007, Australia; 2 Natural Products for Neuroprotection and Anti-Ageing Research Unit, Chulalongkorn University, Bangkok 10330, Thailand; 3 College of Public Health Sciences, Chulalongkorn University, Bangkok 10330, Thailand; 4 Department of Clinical Chemistry, Faculty of Allied Health Sciences, Chulalongkorn University, Bangkok 10330, Thailand

**Keywords:** *Acacia saligna*, polarity-based extraction, bioactivity-guided fractionation, α-glucosidase inhibitors, antioxidants, naringenin-7-O-α-*L*-arabinopyranoside, (3S*,5S*)-3-hydroxy-5-(2-aminoethyl)-dihydrofuran-2(3*H*)-one

## Abstract

*Acacia saligna* growing in Australia has not been fully investigated for its bioactive phytochemicals. Sequential polarity-based extraction was employed to provide four different extracts from individual parts of *A. saligna*. Bioactive extracts were determined using in vitro antioxidant and yeast α-glucosidase inhibitory assays. Methanolic extracts from barks, leaves, and flowers are the most active and have no toxicity against 3T3-L1 adipocytes. Compound isolation of bioactive extracts provided us with ten compounds. Among them are two novel natural products; naringenin-7-O-α-*L*-arabinopyranoside **2** and (3S*,5S*)-3-hydroxy-5-(2-aminoethyl) dihydrofuran-2(3*H*)-one **9**. *D*-(+)-pinitol **5a** (from barks and flowers), (−)-pinitol **5b** (exclusively from leaf), and 2,4-di-*t*-butylphenol **7** are known natural products and new to *A. saligna*. (−)-Epicatechin **6**, quercitrin **4**, and myricitrin **8** showed potent antioxidant activities consistently in DPPH and ABTS assays. (−)-Epicatechin **6** (IC_50_ = 63.58 μM)**,** *D*-(+)-pinitol **5a** (IC_50_ = 74.69 μM), and naringenin **1** (IC_50_ = 89.71 μM) are the strong inhibitors against the α-glucosidase enzyme. The presence of these compounds supports the activities exerted in our methanolic extracts. The presence of 2,4-di-*t*-butylphenol **7** may support the reported allelopathic and antifungal activities. The outcome of this study indicates the potential of Australian *A. saligna* as a rich source of bioactive compounds for drug discovery targeting type 2 diabetes.

## 1. Introduction

*Acacia saligna* (Labill.) H. L. Wendl. is a Western Australian species previously known as *A. cyanophylla* Lindl [1]. *A. saligna* plants growing in Saudi Arabia, Egypt, Tunisia, and other parts of Africa have been shown to have various bioactive phytochemicals. Flavonoids from the flower were shown to have antifungal, antioxidant, antiacetylcholinesterase, and antibacterial activity [2,3,4]. Volatile phytochemicals of *A. saligna*, possessing allelopathic activity, indicate their potential to be green herbicides [5,6]. The leaf extracts containing polyphenols have demonstrated antibacterial and antifungal activities, while some isolated compounds have exhibited antioxidant and cytotoxicity against liver cancer cells [7,8,9]. Two recent reports on the ethanolic crude extract of barks showed antifungal and antioxidant activities [10], and α-glucosidase inhibitory activity [11]. *A. saligna* has also been used in the Middle East, Africa, and South America as ruminants’ fodder [12,13,14]. The utility of *A. saligna* as animal feed indicates its low toxicity and high nutritional benefit.

There is no detailed study on isolating bioactive chemicals from the bark, leaves, and flowers of Australian *A. saligna.* Therefore, this warrants a detailed chemical analysis of beneficial bioactive molecules of this plant.

The literature also indicates that the potential of *A. saligna* growing in Australia has not been fully explored for antidiabetic properties for treating type 2 diabetes (T2D) [11,15]. T2D is a disorder driven by metabolic abnormalities connected to obesity and impaired insulin response, together with the rise of hepatic glucose production, ultimately disrupting glucose homeostasis [16,17]. Previous studies have shown that compounds with antioxidant activity can improve glycaemic control in animal models of T2D [18]. Furthermore, one known therapy for T2D is the application of *α*-glucosidase inhibitors such as acarbose, voglibose, and miglitol. These synthetic inhibitors are known to have adverse side effects, namely hepatotoxicity and gastrointestinal symptoms [19]. It would be beneficial to find safer and more affordable alternatives. Studies have revealed that the α-glucosidase inhibitors and antioxidants could be obtained from natural products such as flavonoids [20]. Therefore, antioxidant and α-glucosidase inhibitory activities would be useful to guide the selection of the most promising extracts for the isolation of pure bioactive compounds as a starting point for developing antidiabetic compounds from Australian *A. saligna*.

In this study, extracts from flowers, leaves, and bark of Australian *A. saligna* were prepared using a sequential polarity-based extraction method. This approach allowed compounds of similar polarity and structure to be pooled into a fraction, which could also streamline pure compound isolation by isolating fewer compounds in the mixture. The process has been known to improve the mass recovery of each pool of compounds [21,22]. The extracts were assessed for antioxidant activity and inhibition against the yeast α-glucosidase. The most bioactive extracts were selected for bioactive compound isolation and complete structure elucidation. Pure compounds’ activities were determined to confirm which were responsible for the activities observed in the extracts.

## 2. Results and Discussion

### 2.1. Fractionation of Crude Extracts of A. saligna

Dried ground powder (250 g) of either flowers, leaves, or bark were sequentially extracted using sequential steps adapted from the protocol of Subhan [23], as shown in Supplementary Figure S1, to give respective extracts as listed in Table 1. The fractionation of each extract was carried out only once; thus, the mass recovery values are presented without standard errors.

### 2.2. Antioxidant Evaluation of Fractions

#### 2.2.1. DPPH Scavenging Activity

**Figure 1:**
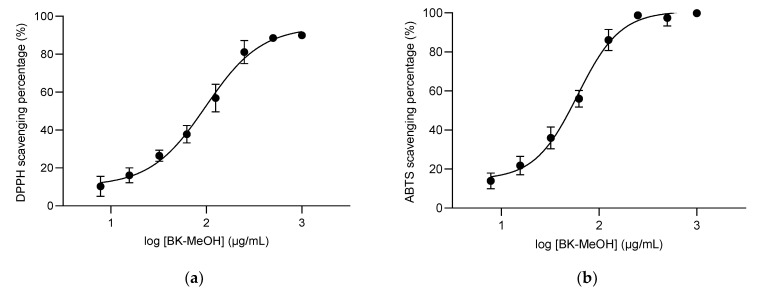
(**a**) The dose-response curve of the inhibitory activity of BK-MeOH against DPPH, (**b**) The dose-response curve of the inhibitory activity of BK-MeOH against ABTS^●+^. Each point represents the average of triplicate measurements.

The dose-response DPPH scavenging activities of all extracts from flowers, leaves, and bark expressed in percentage of activity and IC_50_ are presented in Supplementary Tables S1 and S2 for vitamin C. Figure 1a displays the dose-response curves of the most active BK-MeOH extract. Table 2 shows the IC_50_ value of DPPH scavenging of each extract. The BK-MeOH extract (IC_50_ = 94.24 ± 19.89 µg/mL) has the highest antioxidant activity, followed by LF-MeOH (IC_50_ = 190.1 ± 59.15 µg/mL) and FL-MeOH (IC_50_ = 331.5 ± 17.21 µg/mL). Compared to vitamin C (49.97 ± 10.76 µg/mL), the decreasing antioxidant activities of the methanolic extracts can be expressed as BK-MeOH > LF-MeOH > FL-MeOH. This trend agrees with the previously reported finding that polar organic solvent extract seemed to have better antioxidant activity due to its high polyphenols content [24].

Using a similar DPPH method, the crude ethyl acetate extract from flowers collected in Tunisia was shown to have an IC_50_ of 67 µg/mL [2], while the water flower extract from Egyptian *A. saligna* showed poor activity with an IC_50_ of 461.7 µg/mL [3]. Elansary et al. [4] showed that their crude methanolic extract of leaves collected in Saudi Arabia has a potent antioxidant activity with an IC_50_ of 17 µg/mL. The crude methanolic extract from barks collected in Egypt was reported [10] to have an IC_50_ of 10.1 µg/mL. The variation in activities may mainly be attributed to each extract’s different chemical compositions affected by the growing conditions [25] and methods of extraction and assay.

#### 2.2.2. ABTS^●+^ Radical Assay

The dose-response ABTS^●+^ radical scavenging activities of all extracts are summarised in Supplementary Tables S3 and S4 for vitamin C-positive control. Figure 1b displays the dose-response curves of ABTS^●+^ scavenging percentage for BK-MeOH extract. The IC_50_ values for the three extracts are listed in Table 2. Similar to the DPPH scavenging activity, the trend of antioxidant activities in this ABTS radical assay indicates that all methanolic extracts exert higher activities than their counterparts, compared to vitamin C. The decreasing antioxidant activities of the methanolic extracts can be expressed as BK-MeOH > LF-MeOH > FL-MeOH, compared to vitamin C. Interestingly, BK-MeOH was slightly more active than vitamin C against ABTS^●+^ radical.

### 2.3. Inhibition of Yeast A-Glucosidase Enzyme Assay

The dose-response inhibitory activities of the plant extracts and the positive control acarbose against the yeast α-glucosidase enzyme are shown in Supplementary Tables S5–S8 and Figures S2–S5. The detectable IC_50_ values are listed in Table 3. Non-alcoholic extracts, except aqueous barks extract (BK-H_2_O), are less active than the methanolic samples. BK-MeOH extract showed superior inhibitory activity (IC_50_ 4.373 ± 0.24 µg/mL) compared to BK-H_2_O and the two methanolic counterparts. This value is comparable with the inhibitory activity of crude ethanolic bark and leaf extracts of the South African *A. saligna* with IC_50_ of 2.35 µg/mL and 3.64 µg/mL, respectively [11]. Notably, the inhibitory activity of crude ethanolic leaf extract is more potent than our LF-MeOH.

### 2.4. Toxicity of Bioactive Methanolic Fractions against 3T3-L1 Adipocytes

**Figure 2:**
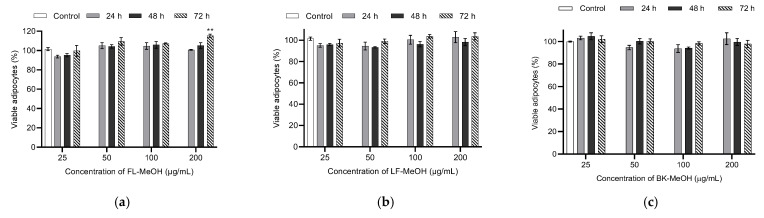
Bar charts of the percentage of viability of 3T3-L1 adipocytes treated with (**a**) FL-MeOH, (**b**) LF-MeOH, and (**c**) BK-MeOH extracts (ANOVA, Dunnett, *n* = 3, ** *p =* 0.003 of treatment with FL-MeOH for 72 h against the vehicle control (0.1% DMSO)).

FL-MeOH, LF-MeOH, and BK-MeOH were tested for their toxicity against 3T3-L1 adipocytes using the 3-(4,5-dimethylthiazol-2-yl)-2,5-diphenyl-tetrazolium bromide (MTT) assay. The 3T3-L1 adipocytes were used in this study because they are the ideal cell model suitable for investigating the antidiabetic activities of *A. saligna.* The cell line can provide an excellent model of white adipose tissue to investigate glucose uptake, lipogenesis, and glycogen synthesis under an insulin-resistant state [26]. Figure 2a shows the results of the MTT assay on 3T3-L1 adipocytes treated with four different concentrations of FL-MeOH (25–200 μg/mL) for 24, 48, and 72 h. The cell viability was estimated between 93 and 116%. FL-MeOH showed no toxic effects at the highest test concentration (200 μg/mL). Similarly, LF-MeOH and BK-MeOH also showed no toxicity against 3T3-L1 adipocytes at 200 μg/mL after incubation for 72 h (Figure 2b,c).

Elansary et al. [4] reported the non-cytotoxic activity of their crude methanolic extract of *A. saligna* leaves from Saudi Arabia against HEK-293 (non-cancerous human embryonic kidney cells). Their MTT assays revealed no significant toxicity against HEK-293 cells at the highest test concentration of 400 μg/mL. Buttner et al. [11] also reported the non-toxicity of their leaf and bark extracts of *A. saligna* from Saudi Arabia against Caco-2 cells at the highest test concentration of 300 mg/mL. Although our non-toxic results against murine 3T3-L1 cells cannot be directly compared to these reported results; however, their findings have highlighted the non-toxicity potential of *A. saligna*.

### 2.5. Isolation of Pure Compounds Active Fractions

**Figure 3:**
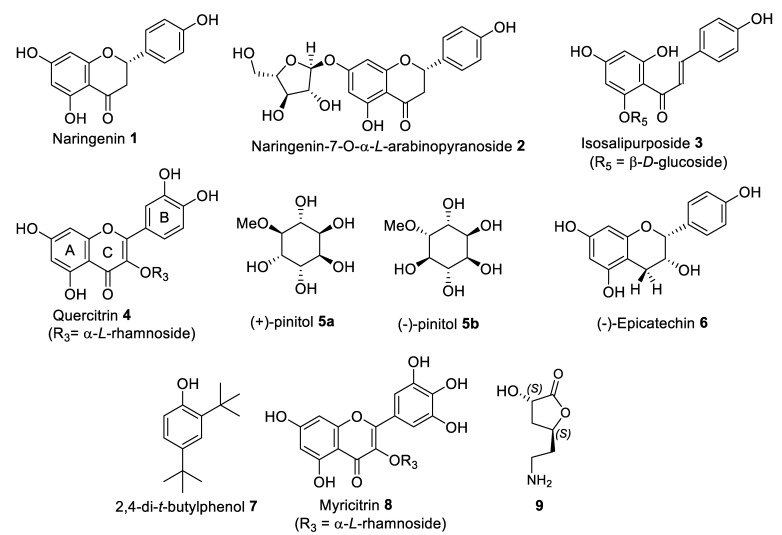
Isolated compounds from FL-OH, LF-OH, and BK-OH extracts.

The most bioactive extracts were selected for compound isolation by column chromatography. Isolated compounds were structure elucidated by NMR and HRMS analysis. We have successfully isolated ten compounds through this effort, as shown in Figure 3. Among these, compounds **2** and **9** are novel. Their isolation and identification are discussed in the following sections.

#### 2.5.1. Isolation and Structural Identification of Compounds from FL-MeOH

FL-MeOH has been prioritised for pure compound isolation because of its superior biological activities and non-toxicity. Two successive column purification of FL-MeOH (Supplementary Figure S6) was performed to give five pure fractions.

Fraction FL-MeOH-A1 is a yellow solid. Complete spectral data analysis of FL-MeOH-A1 revealed that it is naringenin **1** (Figure 3). The NMR data provided in Supplementary Table S9 agrees with that reported in the literature [27,28]. The work by Al-Huqail et al. only reported the presence of naringenin **1** in the water extract of *A. saligna* flowers via their HPLC analysis. Although naringenin **1** has been isolated from other plants [29], it is the first time that we have isolated pure naringenin **1** from the flowers of Australian *A. saligna*. Our study showed that the specific optical rotation of naringenin 1 was ${\left[\alpha \right]}^{23}$= −16.68° (c 0.1, EtOH), which is slightly lower than the previous report ${\left[\alpha \right]}^{22}$= −14.7° (c 0.36, EtOH). This finding confirms the stereochemistry of laevorotatory (−)- or (2*S*)-flavanone comparable to the reported naringenin **1** [28].

**Figure 4:**
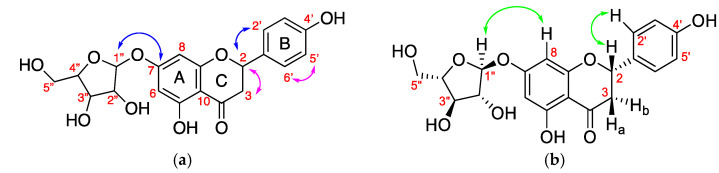
(**a**) key COSY (↔) proton-to-proton connection and HMBC (↔) correlation, (**b**) NOESY (↔) information of naringenin-7-O-α-*L*-arabinopyranoside **2**.

Fraction FL-MeOH-A3 is a bright yellow solid with a melting point of 229–230 °C. The optical rotation of the compound is ${\left[\alpha \right]}^{23}$= −44.54° (c 0.1, MeOH). Detailed NMR analysis of FL-MeOH-A3 is shown in Table 4. The ^13^C NMR chemical shifts of the core skeleton of the compound are identified as a naringenin-related core structure [29]. The following data confirm the typical proton peaks of flavanone. An ABX-system comprises the geminal proton of H3a (2.606 ppm, td, *J* = 17.64, 2.95 Hz, 1H) and H3b (2.946 ppm, m, 1H) coupled to proton H2 (5.255 ppm, td, *J* = 12.96, 2.8 Hz, 1H) in ring C assigned by COSY relationship (Figure 4a). The *p*-substituted aromatic ring B is represented by two stronger downfield signals of δ 7.223 ppm (dd, *J* = 8.56, 3.16 Hz, H2′ and H6′) and δ 6.735 ppm (dd, *J* = 8.64, 1.96 Hz, H3′ and H5′). The catechol ring A was identified by tiny upfield signals for aromatic at δ 6.406 ppm (d, *J* = 2.2 Hz, H8) *o*-coupled to δ 6.059 ppm (d, *J* = 2.24 Hz, H6).

^13^C NMR signals of the sugar moiety are similar to those reported for arabinopyranoside structure by Zhang et al. [30]. The anomeric proton of δ 4.694 ppm (d, *J* = 7.48 Hz, H1″) represented an α-conformation with smaller *J* constants due to the equatorial-axial relationship of H1″ to H2″. The triplet couple of H3″ (3.375 ppm, *J* = 8.4 Hz) and H4″ (3.424 ppm, *J* = 8.08 Hz) with larger *J* constants represents the equatorial-equatorial relationship as an indication of *L*-orientation of the sugar moiety. The geminal protons of 3.876 ppm (m, H5a″) and 3.686 ppm (m, H5b″) as the typical signals for a furanose ring. TOCSY NMR experiment confirmed that the sugar is a 5-membered ring.

The attachment of the sugar moiety at 7-*O* of aromatic ring A was confirmed by C1″-C7 connectivity observed in the 2D HMBC NMR experiment (Figure 4a). Moreover, NOESY NMR (Figure 4b) showed strong cross-peaks between H1″ of the sugar and H8 of aromatic ring A, representing a closer space configuration. The IR spectra gave prominent bands at 3300.70 (OH stretching), 2920.64 (sp^3^ CH stretching), 1605.11 (C=O carbonyl stretching), 1515.07 (C=C aromatic stretching), and 1021.40 cm^−1^ (C–O bending). HRMS showed *m*/*z* [M + H]^+^ of 405.1189 with corresponding molecular formula of C_20_H_20_O_9_ (calculated *m*/*z* = 405.1186). Our spectral data analysis, therefore, concludes FL-MeOH-A3 to be naringenin-7-O-α-L-arabinopyranoside **2** (Figure 4b), a novel flavanone glycone to be isolated from the flowers of Australian *A. saligna*.

FL-MeOH-B1b was isolated as a yellow powder. A complete spectral data interpretation (Supplementary Table S10) of FL-MeOH-B1b was confirmed to be isosalipurposide **3** (Figure 3), which agrees with the literature [2,31]. Isosalipurposide **3** was previously isolated from flowers of *A. saligna* by various groups [2,32,33]. FL-MeOH-B2b was obtained as a yellow powder. Its complete spectral data analysis of this fraction revealed the structure to be quercetin-3-*O*-rhamnoside, also known as quercitrin **4** (Figure 3). The NMR data of FL-MeOH-B2b (Supplementary Table S11) agrees with that reported in the literature [34]. Quercitrin **4** was previously identified in the leaves [8]; it is noteworthy that this is the first time that quercitrin **4** has been isolated from the flowers of Australian *A. saligna*.

FL-MeOH-B3b was isolated as a white solid. Complete spectral data analysis (Supplementary Table S12) of this fraction revealed a structure identical to those reported for *D*-(+)-pinitol **5a** (Figure 3). The optical rotation of FL-MeOH-B3b was found to be ${\left[\alpha \right]}^{23}$= +44.80° (c 0.1, H_2_O), which is lower than the reported value (${\left[\alpha \right]}^{23}$= +65° (c 0.4, H_2_O) [35]. This inositol ether was first isolated in the sugar pine (*Pinus lambertiana*) and could occur in various plants with both enantiomers [36,37]. It was first documented in the related genus *A. nilotica* [38]. However, it has never been isolated as a single enantiomer of pinitol from *A. saligna* flower until now. The compound has been reported as an antidiabetic compound [39,40].

Fractions FL-MeOH-A2, -B1a, -B2a, -B3a, and -C (Supplementary Figure S6) were not further purified due to their low quantities and high impurities.

#### 2.5.2. Isolation and Structural Identification of Compounds from LF-MeOH

Pure compound isolation of LF-MeOH (1000 mg) was carried out with the outcome shown in Supplementary Figure S7 to give six pure fractions.

LF-MeOH-A1 is a colourless solid. Complete spectral data analysis of the compound revealed that it is (−)-epicatechin **6** (Figure 3), which has spectroscopic data (Supplementary Table S13) identical to that reported [41]. The isolation of compound **6** from the leaves of *A. saligna* was previously reported by E-Toumy et al. [7]. However, this study did not provide the compound’s specific optical rotation and absolute stereochemistry. In our work, the optical rotation of LF-MeOH A1 was determined to be ${\left[\alpha \right]}^{23}$= −69.6 °C (c 0.1, MeOH), identical to the literature value [41] of (−)-epicatechin **6** isolated from other plants.

LF-MeOH-A3 is a yellow solid identified by complete spectral data analysis (Supplementary Table S14) as 2,4-di-*t*-butylphenol **7** [42,43] (Figure 3). This compound has been known as a natural toxin and isolated from different groups of organisms, including plants, e.g., sweet potatoes [42] and pine trees [44]. However, for the first time, our group isolated it from the leaves of *A. saligna*. It has also been identified as an antioxidant [45], anticancer, antiviral [46], antibacterial, and antifungal [43]. Moreover, as 2,4-di-*t*-butylphenol **7** demonstrated allelopathic activities against weeds and lettuces [47,48], this compound could also be linked to the reported herbicide properties of *A. saligna*. Indeed, previous works have demonstrated the strong allelopathic [5,49,50] and antifungal activities [4] of *A. saligna* leaves. However, none of those reports identified 2,4-di-*t*-butylphenol **7** and tested its potential.

LF-MeOH-B2 is a yellow powder, similar to FL-MeOH-B2b, and was fully confirmed by spectral data to be quercitrin **7** (Figure 3).

The LF-MeOH-C2b is a yellow powder. Complete spectral data analysis revealed that C2 is myricetin-3-*O*-rhamnoside (Myricitrin) **8** (Figure 3). NMR data listed in Supplementary Table S14 are identical to those reported in the literature [51].

The LF-MeOH-C3 was isolated as a white solid. Complete NMR and HRMS analysis initially revealed that this compound is identical to *D*-(+)-pinitol **5a.** However, its optical rotation ${\left[\alpha \right]}^{23}$= −79.44° (c 0.1, H_2_O) indicates that LF-MeOH-C3 is the enantiomer (−)-pinitol **5b** as shown in Figure 3 ([37] ${\left[\alpha \right]}^{20}$= −61.5° (c 0.19, H_2_O). It is known that both enantiomers of pinitol **5** occur in various plants. However, this is the first time both enantiomers were isolated from Australian *A saligna*. Furthermore, (−)-pinitol **5b** was exclusively isolated from the leaves of this plant.

**Figure 5:**
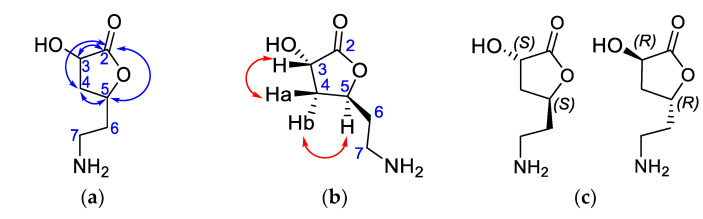
Structural assignment for LF-MeOH-D, **9.** (**a**) HMBC (↔) correlation, (**b**) NOESY (↔) information, and (**c**) two possible conformations of **9**.

LF-MeOH-D was isolated as a colourless solid. HRMS of the compound showed *m/z* [M + H]^+^ 146.0818 corresponding for C_6_H_10_NO_3_ (calculated *m*/*z* = 146.0817). The optical rotation of the compound is ${\left[\alpha \right]}^{23}$= −3.6° (c 0.1, H_2_O). IR showed strong bands at 3212.08 (OH stretch), 2929.09 (CH sp3), 1707.74 (C=O stretch), and 1053.09 cm^−1^ (C–O). Its ^1^H NMR revealed two signals, 1.845 and 4.175 ppm, with integrals of five protons. ^13^C spectrum revealed six carbons, including a carbonyl (δ 175.271 pm), two CH, and three CH_2_ (Table 5). The cross-peaks correlation of TOCSY demonstrated three patterns for (1) H5 to H3 and H4 as well as (2) H5 to H6 and H7; and (3) H4 to H3a and H5. HMBC revealed the key correlations, as shown in Figure 5a. This confirms that a five-membered lactone ring is the core of the compound. The aminoethyl substituent attached to the lactone ring at C5 via C6. The second OH group could be at C4 or C3. The chemical shift of C-3 indicates that the OH is appropriately attached to C3. NOESY NMR analysis (Figure 5b) showed strong crossed peaks between H3-H4a and H5-H4b, indicating that H5 is *trans* to H3. Our spectral data analysis, therefore, concludes LF-MeOH-D to be (*3S,5S*)-3-hydroxy-5-(2-aminoethyl)-dihydrofuran-2(3H)-one **9** or the (*3R,5R*)-enantiomer (Figure 5c). It is for the first time being isolated as a natural product from the leaves of *A. saligna*. Nothing in the literature indicates that compound **9** is a known natural product. The absolute configuration of **9** at C3 and C5 can be further confirmed by an X-ray crystallographic study or NMR analysis of Mosher diastereomeric esters of compound **9**.

Fractions LF-MeOH-A2, -B1, -B3, -C1, C2a, and -C2c (Supplementary Figure S7) were not further purified as they are insufficient in quantity and purity.

#### 2.5.3. Isolation and Structural Identification of Compounds from BK-MeOH

Three main subfractions, BK-MeOH-A1, -B2, and -C2, were isolated (Supplementary Figure S8) from BK-MeOH (300 mg). The NMR data of BK-MeOH-A1 (2.53% *w*/*w*) were identified as (−)-epicatechin **6** (Figure 3). BK-MeOH-B2 (17.83% *w*/*w*) is the major component isolated from BK-MeOH, which was assigned to be *D-*(+)-pinitol **5a**. To our surprise, a disaccharide, sucrose, was identified as the component of BK-MeOH-C2 (8.33% *w*/*w*) by NMR spectral analysis (Supplementary Table S16). The discovery of *D*-(+)-pinitol **5a** in the flowers and the barks and only (−)-pinitol **5b** in the leaves of *A. saligna* is intriguing. In summary, the presence of these compounds is new from the barks of Australia *A. saligna*.

### 2.6. Biological Activities of Isolated Compounds

#### 2.6.1. Scavenging Activities of Isolates against DPPH and ABTS Free Radicals

Out of 10 isolated compounds, only (−)-epicatechin **6**, quercitrin **4**, and myricitrin **8** showed consistently potent antioxidant activities against DPPH and ABTS free radicals (Table 6). On the other hand, the IC_50_ of naringenin **1**; naringenin-7-O-α-*L*-arabinopyranose **2**; 2,4-di-*t*-butylphenol **7**, and 3-hydroxy-5-(2-aminoethyl)-dihydrofuran-2(3*H*)-one **9** did not reach 50% at the highest tested concentration (10 mM) in the DPPH assay (Supplementary Table S17).

Naringenin **1**, naringenin-7-O-α-*L*-arabinopyranose **2**, and 2,4-di-*t*-butylphenol **7** demonstrated a weaker neutralising activity against ABTS^●+^ cation radicals (Table 6) than those of the three flavonoid derivatives. The activity of compound **9** was neglectable as its scavenging percentage remains under 50% even up to 10,000 μM. The lacking π-π conjugation OH groups required to donate H or an electron and the possibility of forming radical resonance intermediates [52,53]; that naringenin **1**, compound **2**, **5b**, **7,** and **9** performed poorly and inconsistently across against DPPH and ABTS free radicals (Supplementary Tables S17 and S18).

IC_50_ values reported in the literature obtained from similar DPPH methods; revealed that myricitrin **8** was active with the IC_50_ ranging from 2.8 to 165.75 µM [51,54]. While (−)-epicatechin **6** was active with IC_50_ in the range of 10.8 to 103.4 µM [55,56]. Similarly, the works by Li et al. [57] and Hong et al. [58] showed that quercitrin **4** has IC_50_ values in the range of 4.45 and 107.5 µM. While naringenin **1** was found to have poor activity by Cai et al. [52] with IC_50_ of 2 mM. Isosalipurposide **3** was also shown to be active with an IC_50_ of 81.9 µM in the DPPH assay [2]. These findings, including ours, reiterate that IC_50_ values are considerably variable.

The presence of quercitrin **4** (4.13% *w*/*w*) and perhaps naringenin **1** (1.75% *w*/*w*) and isosalipuposide **3** (1.52% *w*/*w*) in FL-MeOH supports the activity exerted by this extract.

The presence of the three active antioxidants, namely (−)-epicatechin **6** (0.9% *w*/*w*), quercitrin **4** (2.86% *w*/*w*), myricitrin **8** (5% *w*/*w*), logically supports the activity of LF-MeOH observed in both DPPH and ABTS assays. Quercitrin **4** and myricitrin **8** were also found in the leaf extract of Egyptian *A. saligna* [8]. The potent antioxidant activity of the leaf extract reported by Elansary et al. [4] was extensively exerted by many other flavonoids and polyphenols in the extract, as indicated in their HPLC analysis.

BK-MeOH extract is the most active in both DPPH and ABTS assays (Table 2). However, only 2.53% *w*/*w* of active (−)-epicatechin **6** is present in this extract, in which *D*-(+)-pinitol **5a** (17.83% *w*/*w*) is the main component. In this case, the presence of (−)-epicatechin **6** may partly explain the high activity exerted by BK-MeOH in both assays. The inconsistent activities of *D*-(+)-pinitol **5a** found between DPPH and ABTS assays would inadequately support the activity exerted by BK-MeOH. GC-MS analysis of BK-MeOH (Supplementary Table S19) revealed the presence of cinnamic acid, lamitol, *D*-asparagine, and thymidine-5′-monophosphate. However, these compounds do not have the antioxidant activity to support the observed antioxidant activity in BK-MeOH adequately. In comparison, the antioxidant activity of Egyptian crude ethanolic bark extract was reported to be (IC_50_ = 10.1 µg/mL) [10]. The potent activity of their bark extract is attributed to the presence of antioxidant compounds such as naringenin **1**, kaempferol, rutin, gallic acid, vanillin acid, caffeic acid, ferulic acid, and chlorogenic acid.

#### 2.6.2. α-Glucosidase Inhibition of Isolated Compounds

The yeast α-glucosidase inhibitory activities of isolated compounds were determined using the same procedure for the extracts. The dose-response inhibitory activities of isolated compounds are presented in Supplementary Figure S9 and Table S20. The IC_50_ values of isolated compounds are shown in Table 7. (−)-Epicatechin **6** (IC_50_ = 63.58 ± 11.83 μM), *D*-(+)-pinitol **5a** (IC_50_ = 74.69 ± 0.226 μM), naringenin **1** (IC_50_ = 89.71 ± 10.22 μM), isosalipurposide **3** (IC_50_ = 116.5 ± 26.40 μM), (−)-pinitol **5b** (IC_50_ = 164.2 ± 8.362 μM), and quercitrin **4** (IC_50_ = 177.3 ± 11.34 μM) inhibited the enzyme better than compounds **2**, **7**, **8**, and the positive control, acarbose. In comparison, compound **9** showed no inhibition against the enzyme across the range of test concentrations. It is noteworthy that *D*-(+)-pinitol is a potent inhibitor and is 2-fold more active than its enantiomer (−)-pinitol **5b**. It is important to note that acarbose has been reported to exert more inhibitory activity against mammalian α-glucosidase enzyme than the yeast enzyme. Pacillia et al. [59] reported that naringenin **2** displayed an effective inhibition against the yeast enzyme (IC_50_ 6.51 μM). However, it was poorly active (IC_50_ 384 μM) when tested on the rat intestinal glucosidase. They also reported that the positive control acarbose inhibited the rat α-glucosidase more effectively than the yeast enzyme. Therefore, further investigation is required to confirm the inhibitory activity of our extracts and active compounds against the mammalian α-glucosidase enzyme.

The inhibitory activity of (−)-epicatechin **6** against α-glucosidase was reported to have IC_50_ values in the range of 0.95 µM to 12.3 mM [56,60,61]. For naringenin **3,** variable IC_50_ values were also observed in the range of 6.51 to 75 μM [59,62]. Furthermore, the literature indicates that the reported IC_50_ values of these compounds and other flavonoids are dispersed and variable [63].

The structure-activity relationships (SAR) investigation carried out by Proença et al. [63] suggested that flavonoids with two phenolic groups at the A or B ring and a hydroxy group at C3 possessed the highest α-glucosidase inhibitory activity. He et al. [64] and Şöhretoğlu et al. [20] further reiterated that the number of phenolic groups on ring B is vital for the activity. Their docking study indicated that the B ring of the flavonoids located deep inside the active side of the enzyme and the presence of the phenolics significantly improved interactions via hydrogen bonding. On the other hand, bulky flavonoid glycosides showed poor inhibition due to their inability to access the binding pocket, which explains the poor activity of **2**, quercitrin **4**, myricitrin **8** and **9**. *D*-(+)-pinitol is a cyclic polyol known to have highly beneficial effects on inflammation and related diseases, such as T2D [65]. To the best of our knowledge, it is for the first time that both enantiomers of pinitol were shown to be inhibitors against the yeast α-glucosidase enzyme.

It is noteworthy that *D*-(+)-pinitol **5a** (17.83% *w*/*w*) is the principal component in BK-MeOH and would be the main contributor to the α-glucosidase inhibitory activity observed in the BK-MeOH (IC_50_ = 4.37 ± 0.24 μg/mL) in combination from (−)-epicatechin **6** (2.53% *w*/*w*). BK-H_2_O (IC_50_ = 23.27 ± 3.88 μg/mL) was also active. However, NMR analysis of this fraction revealed that it contains mainly sucrose. Sucrose is a known α-glucosidase substrate [66]; it might outcompete the intended substrate (*p*-nitrophenyl-β-D-glucopyranose, pNPG) of the assay, resulting in the lower concentration of yellow-coloured *p*-nitrophenol cleaved by the enzyme. Therefore, the observed inhibitory activity of BK-H_2_O is more likely associated with a fault-positive inhibition.

(−)-Pinitol **5b** (8% *w*/*w*), (−)-epicatechin **6** (0.9% *w*/*w*), and quercitrin **4** (2.86% *w*/*w*) contribute to inhibitory activity exerted by LF-MeOH (IC_50_ = 38.69 ± 1.01 μg/mL). In FL-MeOH, *D*-(+)-pinitol **5a **(2.5% *w*/*w*), and three mid-range active compounds, namely naringenin **1** (1.75%, *w*/*w*), isosalipurposide **1** (1.52% *w*/*w*), and quercitrin **4** (4.13% *w*/*w*) are the main contributors to the activity found in FL-MeOH (IC_50_ = 34.93 ± 2.67 μg/mL).

## 3. Materials and Methods

### 3.1. General Experimental for Phytochemical Analysis

Isolated pure compounds were structure characterised by specific optical rotation (Jasco P-2000 Polarimeter, Easton, MD, USA), melting point (Gallenkamp apparatus, Apeldoorn, The Netherlands), GCMS (Agilent 6890GC coupled with Agilent 5973n MS (EI), Santa Clara, CA, USA), FTIR (Nicolet-FTIR 6700, Waltham, MA, USA), HRMS (Agilent 6510 QTOF MS (ESI), Santa Clara, CA, USA), 1D and 2D NMR (Bruker 400 MHz, Billerica, MA, USA and Agilent 500 MHz, Santa Clara, CA, USA). Silica gel 60 Å/40–63 µm particle size for flash chromatography, silica gel (SiO_2_) 60 F_254_-coated Thin Layer Chromatography (TLC) aluminium sheets, deuterium oxide (D_2_O), and methanol-d_4_ were supplied by Sigma-Aldrich (St. Louis, MO, USA). The solvents used in the analytical grade were *n*-hexane, methanol, ethyl acetate, dichloromethane, and formic acid.

### 3.2. Sample Collection and Identification

The samples, including leaves, flowers, and stem barks, were collected from 12 Tasman Street, Kurnell, Sutherland Shire, NSW (34°00′48.2″ S 151°12′27.7″ E) on 7 October 2019. The taxonomy of the plant was determined by Andrew Orme (voucher number BIS 21186), a technical identification officer from the National Herbarium of NSW, as *Acacia saligna* (Labill.) H.L.Wendl. The materials were washed with water, air-dried for a week, and finely powdered. The extraction applied sequential extraction with gradually increasing polarity of the solvents: *n*-hexane (hex), dichloromethane (DCM), methanol (MeOH), and water (H_2_O) in a shaker over 48 h at 30 °C, as shown in Supplementary Figure S1.

### 3.3. Sequential Extraction of Plant Parts

The method of extraction was adapted from Subhan [23]. Each extraction was carried out without repetition. Dried flower in powder form (250 g) was first soaked in hexane (1 L) with shaking (80 rpm) at RT for 48 hr, followed by vacuum filtration of the mixture. The resulting filtrate was concentrated under reduced pressure at 35 °C to give FL-hex extract (1.71 g). The solid residue was then air-dried at RT for 12 h and then soaked in dichloromethane (1 L) with the same conditions as above. The resulting filtrate was concentrated under reduced pressure at 35 °C to give FL-DCM extract (1.79 g). The process was repeated for methanol and water to provide the FL-MeOH (26.17 g) and FL-H_2_O (36.31 g). Sequential extraction of dried leaves (250 g) was carried out using the same steps to give LF-hex (3.08 g), LF-DCM (4.98 g), LF-MeOH (25.37 g), and LF-H_2_O (13.32 g), while the sequential extraction of dried barks (250 g) provided BK-hex (0.68 g), BK-DCM (2.12 g), BK-MeOH (18.26 g), and BK-H_2_O (4.34 g), as shown in the Supplementary Figure S1.

### 3.4. General Fractionation Method of Each Methanolic Extract of A. saligna

The methanolic extract from flowers, FL-MeOH (600 mg), was fractionated into three main fractions, namely FL-MeOH-A (109 mg), FL-MeOH-B (206 mg), and FL-MeOH-C (40 mg), as shown in Supplementary Figure S6, with silica gel 60 (Sigma-Aldrich, USA) column chromatography by which and DCM/MeOH (95:5, 90:10, 85:15, and 80:20) was the mobile phase. The selected fraction was then further purified using the same method with a combination of EtOAc/MeOH (100:0, 95:5, and 90:10) eluent system adapted from with a minor modification to afford another three sub-fractions. Successive column purification of fraction A using the gradient EtOAc/MeOH mobile phase gave two pure FL-MeOH-A1 (10.5 mg, 1.75% *w*/*w*) and FL-MeOH-A3 (15.5 mg, 2.58% *w*/*w*). In comparison, FL-MeOH-B provided three pure FL-MeOH-B1b (9.13 mg, 1.52% *w*/*w*) -B2b (24.77 mg, 4.13% *w*/*w*), and -B3b (15 mg, 2.5% *w*/*w*) as shown in Supplementary Figure S6.

### 3.5. Fractionation of LF-MeOH

Pure compound isolation of LF-MeOH (1000 mg) was carried out using the above method, with the outcome shown in Supplementary Figure S7. LF-MeOH-D was isolated in pure form (50 mg, 5% *w*/*w*), while fractions A, B, and C underwent further purification using a gradient EtOH/MeOH eluent system to give five pure sub-fractions: LF-MeOH A1 (9 mg, 0.9% *w*/*w*), LF-MeOH-A3 (10 mg, 1% *w*/*w*), LF-MeOH-B2 (28.6 mg, 2.86% *w*/*w*), LF-MeOH-C2b (50 mg, 5% *w*/*w*) and LF-MeOH-C3 (80 mg, 8% *w*/*w*).

### 3.6. Fractionation of BK-MeOH

BK-MeOH (300 mg) was purified using EtOAc/MeOH (100:0; 95:5, and 90:10) eluent system to give three main fractions (Supplementary Figure S8), BK-MeOH-A1 (7.6 mg, 2.53% *w*/*w*), BK-MeOH-B2 (53.5 mg, 17.83% *w*/*w*), and BK-MeOH-C2 (25 mg, 8.33% *w*/*w*) were isolated.

### 3.7. Spectral Data Analysis

Naringenin **1**: FL-MeOH-A1 (10.5 mg, 1.75% *w*/*w*) is a yellow solid: IR (ν_max_, cm^−1^) 3246.22 (-OH), 2919.77 (sp^3^ CH), 1709.65 (C=O), 1597.51 (C=C aromatic), and 1013.25 cm^−1^ (C–O); HRMS (EI) *m*/*z* 273.0684 [M + H], cald for C_15_H_12_O_5_ 273.0685; m.p. 253–255 °C. ${\left[\alpha \right]}^{23}$= −16.68° (c 0.1, EtOH) [lit. [67] ${\left[\alpha \right]}^{22}$= −14.7 (c 0.36, EtOH)]. For complete NMR analysis, see Supporting information (Supplementary Table S9).

Naringenin-7-O-α-L-arabinopyranoside **2**: FL-MeOH-A3 (15.5 mg, 2.58% *w*/*w*) is yellow powder: IR (ν_max_, cm^−1^) 3300.70 (-OH stretching), 2920.64 (sp^3^ CH stretching), 1710 C=O), 1605.11 (C=C), and 1021.40 cm^−1^ (C–O); HRMS (EI) *m*/*z* 405.1189 [M + H], cald C_20_H_20_O_9_ 405.1186; m.p. 229–230 °C. ${\left[\alpha \right]}^{23}$= −44.54° (c 0.1, MeOH). For a complete NMR analysis, see Table 3.

Isolisalipurposide **3**: FL-MeOH-B1b is a yellow powder (9.13 mg, 1.52% *w*/*w*): IR (ν_max_, cm^−1^) 3255.08 (-OH), 2930.83 (sp^3^ CH), 1601.61 (C=O), 1550.28 (C=C), and 1070.60 cm^−1^ (C–O); HRMS (EI) *m/z* 435.1298 [M + H], cald C_21_H_22_O_10_ 435.1291; m.p. 174–175 °C; ${\left[\alpha \right]}^{23}$= −119.02° (c 0.1, MeOH). For a complete NMR analysis, see Supplementary Table S10.

Quercitrin **4**: FL-MeOH-B2b (24.77 mg, 4.13% *w*/*w*) and LF-MeOH-B2 (28.6 mg, 2.86 *w*/*w*) are the same isolated as yellow solids: IR (ν_max_, cm^−1^) 3248.57 (-OH), 2936.97 (sp^3^ CH), 1652.71 (C=O), 1499.15 (C=C), 1198.56, and 1070.60 cm^−1^ (C–O); HRMS(EI) *m/z* 449.1072 [M + H], cald C_21_H_21_O_11_ 449.1084; m.p. 180–183 °C; ${\left[\alpha \right]}^{23}$= −120.86^o^ (c 0.1, MeOH). For a complete NMR analysis, see Supplementary Table S11.

*D-(+)*-Pinitol **5a**: FL-MeOH-B3b (15 mg, 2.5% *w*/*w*) and BK-MeOH-B2 (53.5 mg, 17.83% *w*/*w*) are the same, isolated as white solids: IR (ν_max_, cm^−1^) 3328.27 (-OH), 2918.85 (sp^3^ CH), and 1034.21 cm^−1^ (C–O); HRMS (EI) *m/z* 195.0865 [M + H]^+^, cald C_7_H_14_O_6_ 195.0869; m.p. 171–172 °C; ${\left[\alpha \right]}^{23}$= +44.80° (c 0.1, H_2_O) [lit. [37] ${\left[\alpha \right]}^{23}$= +69.7° (c 0.56, MeOH)]. For full NMR analysis, see Supplementary Table S12.

(−)-Pinitol **5b**: LF-MeOH-C3 (80 mg, 8%) a white solid: IR (ν_max_, cm^−1^) 3389.09 (-OH), 3302.90 and 2907.93 (sp^3^ CH), 1068.27 and 1070.60 cm^−1^ (C–O); HRMS (EI) *m/z* 195.0863 [M + H], cald C_7_H_14_O_6_ 195.0869; m.p. 175–177 °C; ${\left[\alpha \right]}^{23}$= −79.44° (c 0.1, H_2_O) [lit. [37] ${\left[\alpha \right]}^{20}$= −61.5° (c 0.19, H_2_O)]. For full NMR analysis, see Supplementary Table S12.

(−)-Epicatechin **6:** LF-MeOH A1 (9 mg, 0.9%) and BK-MeOH-A1 (7.6 mg, 2.53%) are the same, isolated as colourless solids: IR (ν_max_, cm^−1^) 3220.47 (-OH); 2919.54 (sp^3^ CH); 1604.73 (C=C); and 1031.13 cm^−1^ (C–O); HRMS (EI) *m/z* 291.0858 [M + H]^+^ cald C_15_H_14_O_6_ 291.0869; m.p. 240–243 °C; ${\left[\alpha \right]}^{23}$= −69.6 ^o^C (c 0.1, MeOH) [lit. [45] ${\left[\alpha \right]}^{23}$= −69.7° (c 0.56, MeOH)]. For full NMR analysis, see Supplementary Table S13.

2,4-di-*t*-butylphenol **7**: LF-MeOH-A3 (10 mg, 1% *w*/*w*) is a yellow solid: IR (ν_max_, cm^−1^) 3330.29 (-OH), 2921.70 (sp^3^ CH), 1592.96 (C=C, aromatic), and 1029.41 cm^−1^ (C–O); HRMS (EI) *m*/*z* 207.1662 [M + H], cald C_14_H_22_O 207.1749; m.p. 61–62 °C. For full NMR analysis, see Supplementary Table S14.

Myricetin 3-*O*-rhamnoside (myricitrin) **8**: LF-MeOH-C2b (50 mg, 5% *w*/*w*) is a yellow powder: IR (ν_max_, cm^−1^) 3266.81 (-OH), 2930.51 (sp^3^ CH), 1652.84 (C=O), 1499.04 (C=C aromatic), 1197.39 and 1070.60 cm^−1^ (C–O); HRMS (EI) *m*/*z* 465.1037 [M + H], cald C_21_H_20_O_12_ 465.1033; m.p. 193–195 °C; ${\left[\alpha \right]}^{23}$= −246.32° (c 0.1, MeOH). For a complete NMR analysis, see Supplementary Table S15.

(3S*,5S*)-3-Hydroxy-5-(2-aminoethyl) dihydrofuran-2(3*H*)-one **9**: LF-MeOH-D (50 mg, 5% *w*/*w*) a colourless solid: IR (ν_max_, cm^−1^) 3212.08 (OH), 2929.09 (sp^3^ CH), 1707.74 (C=O) and 1053.09 (C-O), HRMS (EI) *m/z* 146.0818 [M + H]^+^ cald C_6_H_10_O_4_ 146.0817; m.p. 347–350 °C. ${\left[\alpha \right]}^{23}$= −3.6° (c 0.1, H_2_O). For a full NMR analysis, see Table 4.

Sucrose: BK-MeOH-C2 (25 mg, 4.1% *w*/*w*). For full NMR analysis, see Supplementary Table S16.

### 3.8. Biological Assay

#### Materials

All chemicals used in this assay were of analytical grade. Absolute ethanol was provided by Point of Care Diagnostics (North Rocks, NSW, Australia). Ascorbic acid was purchased from Merck (Darmstadt, Germany). 2,2-di(4-tert-octylphenyl)-1-picrylhydrazyl (DPPH), 2,2′-azino-bis-(3-ethylbenzothiazoline-6-sulphonic acid (ABTS), an α-glucosidase enzyme (EC-No.: 232-604-7) from *Saccharomyces cerevisiae* (lyophilised powder, 23 units/mg), 4-nitrophenyl α-D-glucopyranoside (*p*-NPG, ≥99%), acarbose (99%), Dulbecco’s Modified Eagle’s Medium High Glucose (DMEM), bovine calf serum (BCS), penicillin, streptomycin, glutamine (PSG), fetal bovine serum (FBS), rosiglitazone, dexamethasone, 3-isobutyl-1-methylxanthine (IBMX), insulin, phosphate-buffered saline (PBS), (3-(4,5 dimethylthiazol-2-yl)-2, 5 diphenyltetrazolium bromide) (MTT), trypsin-EDTA solution 0.25%, and dimethylsulfoxide (DMSO) were purchased from Sigma-Aldrich (St. Louis, MO, USA). The 3T3-L1 murine cell line was supplied by American Type Tissue Culture/ATCC (Manassas, VA, USA).

### 3.9. DPPH Scavenging Assay

The DPPH-free radical scavenging study based on a 96-well plate reading approach was performed following Jiang et al. [68] and Chen et al. [69] with slight modifications. Briefly, a 180 µL of DPPH 0.2 mM ethanolic solution was pipetted into each well (Corning, New York City, NY, USA), followed by 20 µL of ethanolic solution of extracts or ascorbic acid in a different concentration and, for the blank solution, 20 µL of ethanol. The blank extract and the blank positive solution were prepared by adding 180 µL of ethanol into 20 µL of samples and ascorbic acid solution. The plate was then incubated in a dark condition for 30 min at 30 °C. The absorbance was observed using a microplate reader (Tecan Infinite M1000 PRO, Männedorf, Switzerland) at 517 nm. The percentage of DPPH scavenging activity was determined by:

$$\mathrm{DPPH}\;\mathrm{scavenging}\;\mathrm{activity}\;\left(\%\right)=\left[1-\left(\frac{A_{1}-A_{2}}{A_{0}}\right)\right]\times 100\%\;$$

*A*_0_ is the absorbance of the blank solution (DPPH 0.2 mM + EtOH), *A*_1_ is the absorbance of the sample (sample + DPPH 0.2 mM), and *A_2_* is the absorbance of the blank sample (sample in EtOH).

The value was then converted into IC_50_ (µg/mL) from a graph correlating the sample concentration (mg/mL) and DPPH scavenging activity (%). The results were expressed as mean ± standard error mean (SEM) of three separate experiments (*n* = 3). The descriptive statistics are analysed in GraphPad Prism 8 (San Diego, CA, USA).

### 3.10. ABTS^●+^ Radical Decolourisation Assay

The ABTS^●+^ solution was prepared by generating a reaction between ABTS 7 mM and potassium persulfate 2.45 mM (1:1 of *v*/*v*) at room temperature for 16–18 h [70]. The ABTS^●+^ solution was further diluted to achieve an acceptable measurement at 734 nm [71]. The same experimental procedure used in the DPPH radical scavenging assay was applied to measure the percentage of ABTS^●+^ radical scavenging. The absorbance was observed using a microplate reader (Tecan Infinite M1000 PRO, Männedorf, Switzerland) at 734 nm.

### 3.11. In Vitro Assay of Yeast α-Glucosidase Inhibition

The enzyme deactivation assay was carried out following the modified microplates method adapted from Ning et al. [72]. A volume of 20 µL of the plant extract in different concentrations or acarbose solution (31.25 to 1000 µg/mL), isolates or acarbose solution (31.25 to 1000 µM), or solvent control was mixed with α-glucosidase (40 µL, 0.075 U/mL in potassium phosphate buffer solution (100 mM, pH 6.8) in 96-well polystyrene plates (Corning, New York City, NY, USA) and then incubated for 15 min at 37 °C. Afterwards, *p*-NPG solution in the buffer solution (40 µL, 1 mM) was added to the mixture, followed by further incubation for 30 min at 37 °C. The reaction was terminated by adding Na_2_CO_3_ solution (100 μL, 200 mM) to the wells. The spectrophotometric observation was then conducted to determine the absorbance of *p*-nitrophenol released from the reaction under 405 nm wavelength in a microplate reader (Tecan Infinite M1000 PRO, Männedorf, Switzerland). The percentage of inhibition was calculated from the following formula:

$$\mathrm{Percentage}\;\mathrm{of}\;\mathrm{inhibition}\;\left(\%\right)=\frac{A_{c}-A_{s}}{A_{c}}\times 100\%\;$$

where *A_c_* is the absorbance of the solvent control and enzymatic reaction system and *A_s_* is the absorbance of the sample with the enzymatic reaction system. The inhibitory activity was expressed in the value of half minimal inhibitory concentration (IC_50_).

### 3.12. Differentiation of 3T3-L1 Preadipocytes into Adipocytes

The 3T3-L1 preadipocytes (70–80% confluent from a culture flask) were grown in a 96-well microtiter plate (3 × 10^3^ cells/well in 100 µL final volume of basal medium 1 (M1 = 90% DMEM, 9% BCS, and 1% PSG) and incubated for 48 h in a humid condition (37 °C& 5% CO_2_) for adherence of the cells. After 48 h, the old M1 was replaced with new M1, and the cells were incubated for another 48 h (day −2 to 0) to get 100% confluent. The M1 was replaced by an identical volume of M2 (9% FBS, 1% PSG, and 90% DMEM containing rosiglitazone 2 µM, dexamethasone 2.2 mM, IBMX 500 mM, and insulin 4 mg/mL) followed by incubation for 48 h (day 0 to 2). After incubation and M2 removal at day 2 of differentiation, new M3 (90% DMEM, 9% FBS, 1% PSG, and insulin) was added, followed by incubation to day 6 with medium replacement every 48 h. On day six, M3 was replaced by M4 (90% DMEM, 9% FBS, and 1% PSG), followed by another 48 h of incubation, as shown in Table 8 below.

### 3.13. Effects of Extracts on the Viability of 3T3-L1 Adipocytes

The differentiated 3T3-L1 cells grown in three 96-well microtiter plates (Corning, New York City, NY, USA) were exposed to 100 μL of fresh test solution containing flower, leaf, and bark extracts in a range of concentration of 25–200 μg/mL and incubated for a further 24, 48, and 72 h. After incubation, the solution was replaced with 100 μL of fresh medium containing 10 % MTT solution (5 mg/mL in PBS). The treated cells were then incubated for an additional 4 h. Once finished the last incubation, the MTT solution was replaced by 100 μL of DMSO to solubilise the formazan crystal products. The absorbance was measured at the wavelength of 570 via a multiwell plate reader (Tecan Infinite M1000 PRO, Männedorf, Switzerland). Each concentration was performed twice times whereby each experiment was conducted in triplicate. The percentage of cell viability is expressed in the:

$$\mathrm{Cell}\;\mathrm{viability}\;(\%)\;=\;\frac{\mathrm{absorbance}\;\mathrm{of}\;\mathrm{sample}}{\mathrm{absorbance}\;\mathrm{of}\;\mathrm{control}}\times \;100\%$$

### 3.14. Statistical Method

The results were expressed as mean ± standard error mean (SEM) of three independent experiments (*n* = 3). The results were analysed using a one-way analysis of variance (ANOVA) with Tukey’s or Dunnett’s post hoc test using GraphPad Prism 8 (Boston, MA, USA). The difference was considered significant at *p* < 0.05.

## 4. Conclusions

Our approach in using sequential polarity-based extraction of *A. saligna* parts and bioactivity-guided fractionation has fast-tracked the identification of bioactive compounds in *A. saligna*. Isolation of pure compounds in the active methanolic extracts was greatly simplified, evidenced by the requirement of one or two successive steps in column chromatography. Through this effort, we have successfully isolated ten compounds of different categories. They are (i) isosalipurposide **3**, myricitrin **8,** and (−)-epicatechin **6** as known compounds isolated from *A. saligna*; (ii) naringenin **1** and quercitrin **4** as known compounds to exist in *A. saligna*; however, being isolated in pure form in this work, (iii) *D*-(+)-pinitol **5a**, (−)-pinitol **5b** and 2,4-di-*t*-butylphenol **7** as known natural products found elsewhere, however, are new to this plant, and (iv) naringenin-7-O-α-*L*-arabinopyranose **2** and (3S*,5S*)-3-hydroxy-5-(2-aminoethyl) dihydrofuran-2(3*H*)-one **9** as two novel natural products. The antioxidant and α-glucosidase inhibitory activities of the isolated compounds, especially (−)-epicatechin **6**, naringenin **1**, and *D*-(+)-pinitol **5b**, quercitrin **4**, and myricitrin **8,** support the activities observed in our methanolic extracts and the reported activities of crude extracts of *A. saligna*. The presence of 2,4-di-*t*-butylphenol **7** may also help to explain the reported allelopathic and antifungal activities of *A. saligna*. The outcome of this study indicates the potential of *A. saligna* as a rich source of bioactive compounds for drug discovery targeting T2D.

## Figures and Tables

**Table 1:** The result of the extraction of the flowers, leaves, and barks of *A. saligna*.

Type of Extract	Mass of Extract (g), Colour		
	Dried Flowers (FL)	Dried Leaves (LF)	Dried Barks (BK)
Hexane (hex)	1.71, yellow	3.08, dark green	0.68, yellow
Dichloromethane (DCM)	1.79, green	4.98, green	2.12, green
Methanol (MeOH)	26.16, yellow	25.37, green-yellow	18.26, yellow
Water (H_2_O)	36.31, yellow	13.32, yellow	4.34, yellow

**Table 2:** Antioxidant activities expressed in IC_50_ between methanolic flowers, leaves, and barks extract of *A. saligna* and vitamin C in DPPH and ABTS^●+^ scavenging assay, **** *p* < 0.0001 of the indicated extracts against vitamin C (*n* = 3, ANOVA, Tukey).

Extracts	IC_50_ (µg/mL)	
	DPPH Assay	ABTS^●+^ Assay
FL-MeOH	331.5 ± 17.21 ****	316.6 ± 11.45 ****
LF-MeOH	190.1 ± 59.15 ****	146.7 ± 0.99
BK-MeOH	94.24 ± 19.89	55.44 ± 6.84
Vitamin C	49.97 ± 10.76	72.25 ± 4.42

**Table 3:** IC_50_ values of active extracts from flowers, leaves, and barks of *A. saligna* and acarbose against yeast α-glucosidase, *** *p* = 0.0004 of FL-MeOH extract vs. acarbose; **** *p* < 0.0001 were inhibition of the LF-MeOH, BK-MeOH, and BK-H_2_O extracts vs. acarbose (*n =* 3, ANOVA, Dunnett).

Extracts	IC_50_ (µg/mL)
FL-MeOH	34.93 ± 2.67 ***
LF-hex	285.5 ± 100.9
LF-MeOH	38.69 ± 1.01 ***
BK-hex	289.9 ± 29.17
BK-MeOH	4.37 ± 0.24 ****
BK-H_2_O	23.27 ± 3.88 ****
Acarbose	254 ± 22.18

**Table 4:** The 1- and 2-D NMR data of naringenin-7-O-α-*L*-arabinopyranoside **2** (FL-MeOH-A3 in CD_3_OD).

ID	^1^H (δ ppm, m, *J* in Hz, Integration)	δ ^13^C (ppm)	COSY	TOCSY	NOESY	HMBC(C→H) ^a^
2	5.35 (td; 12.96, 2.8; 1H)	80.48	H3a H3b	H3a, H3b	H3b (strong), H3a (weak)	C4, C2′, C6′
3a	2.70 (td; 17.64, 2.95; 1H)	46.45	H2, H3b	H2, H3b	H2	C4, C10
3b	3.04 (m; 1H)		H2, H3a	H2, H3a	H2	C4, C2, C1′
4		193.22				
5		166.70				
6	6.15 (d; 2.24; 1H)	99.56	H6	H8	H8	C4, C5, C8 C10
7		167.16				
8	6.50 (d; 2.2; 1H)	100.49	H8	H6	H6	C4, C7, C6, C9, C10
9		162.51				
10		107.21				
1′		131.12				
2′	7.31 (dd; 8.56, 3.16; 1H)	129.14	H3′, H6′	H3′, H6′	H3′ (strong), H2 (weak)	C2, C1′, C3′, C4′
3′	6.83 (dd; 8.64, 1.96; 1H)	116.46	H2′, H5′	H2′, H5′	H2′	C2′, C4′
4′		159.13				
5′	6.83 (dd; 8.64, 1.96; 1H)	116.46	H3′, H6′	H3′, H6′	H6′	C6′, C4′
6′	7.31 (dd; 8.56, 3.16; 1H)	129.14	H2′, H5′	H2′, H5′	H5′	C2, C1′, C5′, C4′
1”	4.78 (d; 7.48; 1H)	105.16	H2″	H2”, H3”, H4”	H8 (strong), H2″, H3″, H4″	C7, C2″
2”	3.57 (m; 1H)	74.81	H1″	H1″, H3″, H4″, H5″a, H5b″	H1″, H5″a	
3”	3.45 (t; 8.4; 1H)	78.79		H1″, H2″, H4″, H5″a, H5b″	H1″, H5″a, H5″b	C2″
4”	3.51 (t; 8.08; 1H)	77.3	H5″b	H1″, H2″, H3″, H5″a, H5b″	H1″, H5″a, H5″b	C2″
5”a	3.97 (m; 1H)	62.68	H5″b	H2″, H3″, H4″, H5b″	H2″, H3″, H4″, H5″b	
5”b	3.78 (m; 1H)		H4″, H5″a	H2″, H3″, H4″, H5a″	H3″, H4″, H5″a	

^a^ HMBC correlations are from carbon(s) stated to the indicated proton (ID).

**Table 5:** The 1- and 2-D NMR data of isolate from LF-MeOH-D in D_2_O (400 MHz).

ID	1H (δ ppm, m, J Hz, Integration)	13C (δ ppm)	COSY	TOCSY	HMBC(C→H) ^a^	NOESY
2		175.27				
3	3.86 (dd; 11.8, 3.68; 1H)	53.90	H4b, H4b	H5, H4a, H4b	C2, C4, C5	H4a (strong), H4b
4a	2.16 (m; 1H)	32.74	H3, H4b, H5	H3, H5, H4b		H3 (strong), H4b (strong)
4b	1.91 (m; 1H)		H3, H4a, H5	H3, H5, H4a	C2, C3, C5, C6	H5 (strong), H4a
5	4.18 (m; 1H)	61.80	H4a, H4b, H6	H3, H4a, H4b, H6, H7		H4b (strong), H6 (strong)
6	1.85 (m; 2H)	26.50	H5, H7	H5, H7	C4, C5, C7	H5
7	3.26 (m; 2H)	38.54	H6	H5, H6	C5, C6	H6 (strong)

^a^ HMBC correlations are from carbon(s) stated to the indicated proton (ID).

**Table 6:** The DPPH and ABTS scavenging properties of the isolated compounds.

Compounds	IC_50_ against DPPH (µM)	IC_50_ against ABTS (µM)
Isosalipurposide **3**	1559 ± 28.16 ***	1686 ± 95.26 ****
Naringenin **1**	>10,000 ^a^	1525 ± 316.50 ****
Quercitrin **4**	322.6 ± 14.05 ****	355.3 ± 12.08
Myricitrin **8**	199.9 ± 4.83 ****	285.9 ± 7.21
Naringenin-7-O-α-*L*-arabinopyranose **2**	>10,000 ^a^	4146 ± 99.15 ****
*D*-(+)-pinitol **5a**	1675 ± 65.72 ****	475 ± 24.20
(−)-Pinitol **5b**	6865 ± 69.08 ****	2096 ± 70.40 ****
(−)-Epicatechin **6**	278 ± 8.62 ****	92.58 ± 13.03
2,4-Di-*t*-butylphenol **7**	>10,000 ^a^	2715 ± 64.02 ****
3-hydroxy-5-(2-aminoethyl)-dihydrofuran-2(3*H*)-one **9**	>10,000 ^a^	>10,000 ^a^
Vitamin C	1072 ± 47.64	460.2 ± 56.29

^a^ The activity did not reach 50% at the highest tested concentration (10 mM); *** *p =* 0.0002 of compound 3 vs. vitamin C; **** *p <* 0.0001 were from the indicated compounds vs. vitamin C (*n* = 3, ANOVA, Tukey).

**Table 7:** Inhibitory activity of the isolated compounds against yeast α-glucosidase.

Compound	IC_50_ (μM)
Isosalipurposide **3**	116.5 ± 26.40
Naringenin **2**	89.71 ± 10.22 *
Quercitrin **4**	177.3 ± 11.34
Myricitrin **8**	351.6 ± 24.88
Naringenin-7-O-α-*L*-arabinopyranose **2**	769.1 ± 95.82 ****
*D*-(+)-pinitol **5a**	74.69 ± 0.23 *
(−)-pinitol **5b**	164.2 ± 8.36
(−)-Epicatechin **6**	63.58 ± 11.83 *
2,4-Di-*t*-butylphenol **7**	259 ± 58.34
3-hydroxy-5-(2-aminoethyl) dihydrofuran-2(3*H*)-one **9**	>1000 ^a^
Acarbose	239.9 ± 31.74

^a^ The activity did not reach 50% at the highest tested concentration (1 mM); * *p =* 0.03, **** *p <* 0.0001 were from the inhibition of the compound vs. acarbose (*n =* 3, ANOVA, Tukey).

**Table 8:** Schematic workflow diagram of the cell differentiation.

Cell growth		100% confluent	Lipid droplets formation and development			Excessive lipid droplets
48 h	48 h (day −2 to 0)	48 h (day 0 to 2)	48 h (day 2 to 4)	48 h (day 4 to 6)	48h (day 6 to 8)	Between day 8 & 12
Feed *M1*	Feed *M1*	Feed *M2*	Feed *M3*	Feed *M3*	Feed *M4*	Treatment with extracts/compounds
**Preadipocyte** 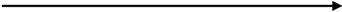 **mature adipocyte**						

## Data Availability

Data is contained within the article.

## Supplementary Materials

The following supporting information can be downloaded at: https://www.mdpi.com/article/10.3390/molecules28031028/s1. Figure S1: Sequential extraction of *A. saligna*’s plant parts; Tables S1 and S2: DPPH scavenging activity of the extracts of *A. Saligna*; Tables S3 and S4: ABTS^●+^ scavenging activity of the extracts of *A. Saligna*; Table S5a,b & Figure S2: Percentage of α-glucosidase inhibition (%) of FL-MeOH; Table S6a,b & Figure S3: Percentage of α-glucosidase inhibition (%) of LF-MeOH; Table S7a,b & Figure S4: Percentage of α-glucosidase inhibition (%) of BK-MeOH; Table S8 & Figure S5: Percentage of α-glucosidase inhibition (%) of acarbose; Figure S6: Schematic fractionation of FL-MeOH extract of *A. saligna*; Table S9: NMR data of FL-MeOH-A1 compared to reported naringenin **1**; Table S10: NMR data of FL-MeOH-B1b compared to reported isosalipurposide **3**; Table S11: NMR data FL-MeOH-B2b compared to reported quercitrin 4; Table S12: NMR data of FL-MeOH-B3b compared to reported D-pinitol 5a; Figure S7: Schematic of fractionation of LF-MeOH extract of *A. saligna*; Table S13: NMR data of LF-MeOH-A1 compared to reported (-)-epicatechin **6**; Table S14: NMR data of LF-MeOH-A3 compared to 2,4-di-t-buylphenol **7**; Table S15: NMR data of LF-MeOH-C2b compared to reported myricitrin **8**; Figure S8: Schematic fractionation of BK-MeOH extract of *A. saligna*; Table S16: NMR data of BK-MeOH-C compared to reported sucrose; Table S17: The DPPH scavenging properties of the isolated compounds; Table S18: The ABTS^●+^ scavenging properties of the isolated compounds; Table S19: GC analysis of BK-MeOH; Figure S9: Inhibitory activity of isolated compounds against the α-glucosidase; Table S20: Inhibitory activity of the isolated compounds against the α-glucosidase enzyme.

## Sample Availability

Samples of the compounds are not available from the authors.
