# Supplementary material for: Identification of Phytochemicals in Bioactive Extracts of Acacia saligna Growing in Australia
Source: Molecules. 2023 Jan 19;28(3):1028. doi: 10.3390/molecules28031028 (PMC9919957; doi:10.3390/molecules28031028)
Supplement: Supplementary file 1 [file molecules-28-01028-s001.zip › molecules-2141069-supplementary.pdf]

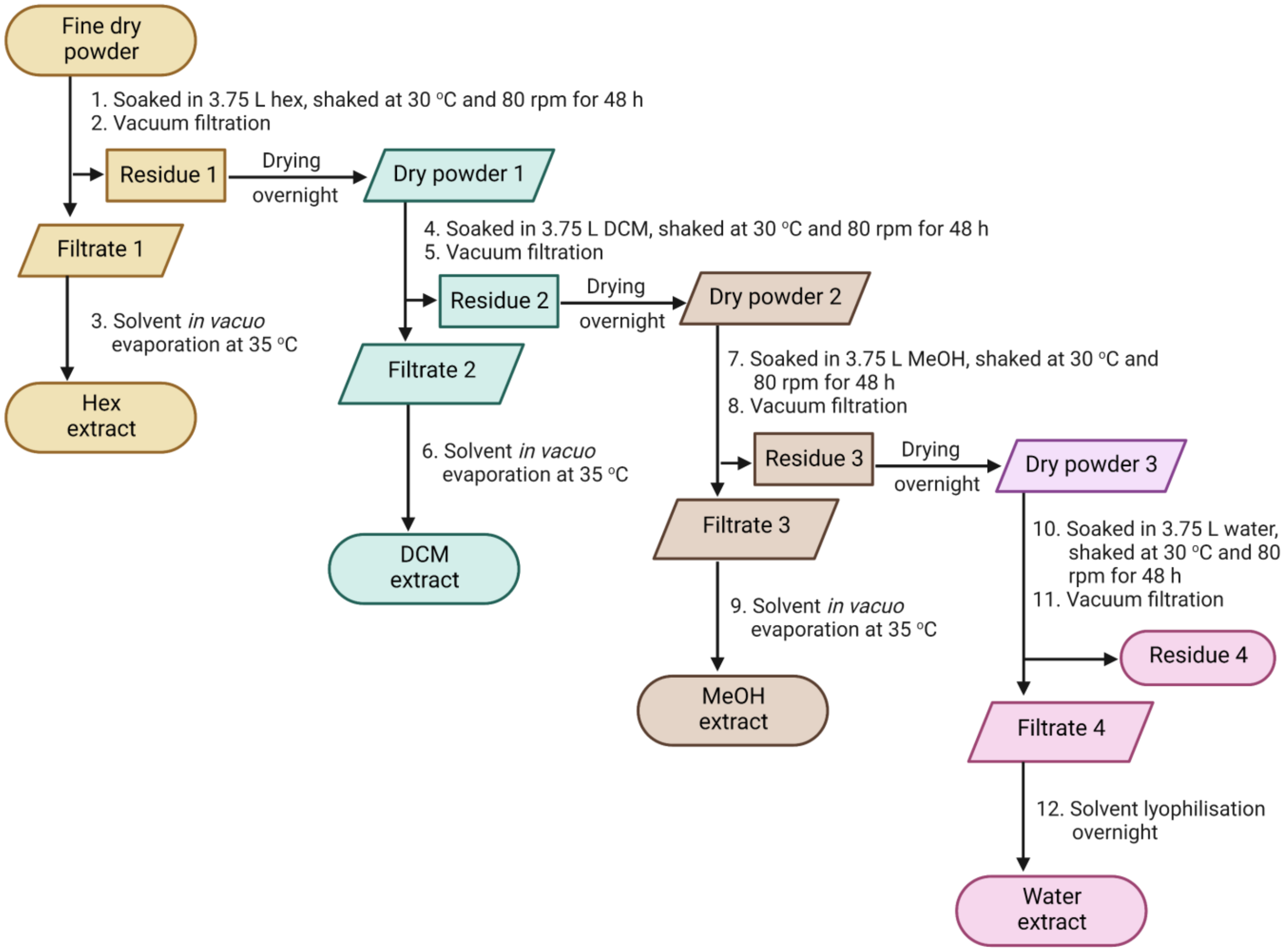

Figure S1. Illustration of the sequential extraction of *A. saligna's* plant parts (Note: Hex = hexane, DCM= dichloromethane, MeOH = methanol)

Table S1. DPPH scavenging activity of the extracts of *A. saligna*

No	Extract	DPPH scavenging percentage (%) at the indicated concentration (µg/mL)										IC_{50} (µg/mL)
		7.8125	15.625	31.25	62.5	125	250	500	1,000	2,000	4,000	
1	FL-hex	-	-	6.306 ± 3.09	7.537 ± 2.97	12.19 ± 4.03	10.47 ± 4.30	11.07 ± 4.56	11.5 ± 6.78	13.64 ± 6.44	19.1 ± 6.40	>4000[a]
2	FL-DCM	-	-	0.645 ± 6.16	3.976 ± 6.61	2.068 ± 7.92	3.639 ± 9.03	8.673 ± 6.85	15.91 ± 5.14	14.36 ± 5.94	22.87 ± 3.57	>4000[a]
3	FL-MeOH	8.67 ± 5.44	8.05 ± 4.06	9.32 ± 1.461	16.48 ± 0.91	27.8 ± 0.03	41.57 ± 1.3	61.07 ± 1.30	74.34 ± 0.87	-	-	331.5 ± 17.21****
4	FL-H_2O	-	-	1.07 ± 2.96	2.54 ± 2.95	4.32 ± 3.34	5.45 ± 4.09	10.51 ± 2.65	18.09 ± 3.42	20.92 ± 2.11	34.78 ± 5.75	>4000[a]
5	LF-hex	-	-	10.04 ± 10.46	12.43 ± 8.05	18.1 ± 5.99	18.82 ± 3.36	18.96 ± 8.98	22.26 ± 7.29	34.45 ± 9.91	60.47 ± 11.55	3,283 ± 774.3****
6	LF-DCM	-	-	1.391 ± 10.24	4.17 ± 15.54	3.49 ± 9.81	5.7 ± 8.78	14.81 ± 7.81	27.72 ± 7.85	24.3 ± 7.28	37.41 ± 7.09	>4000[a]
7	LF-MeOH	10.75 ± 6.58	13.33 ± 7.44	21.33 ± 7.21	25.89 ± 7.56	38.66 ± 8.94	58.2 ± 9.95	82.6 ± 6.20	89.33 ± 0.61	-	-	190.1 ± 59.15****
8	LF-H_2O	-	-	3.61 ± 2.90	5.06 ± 3.31	7.55 ± 4.40	8.12 ± 6.56	14.03 ± 7.4	26.06 ± 5.18	31.14 ± 5.65	47.9 ± 5.08	>4000[a]
9	BK-hex	-	-	2.27 ± 7.00	5.57 ± 6.03	12.39 ± 2.51	10.06 ± 3.23	9.764 ± 4.26	11.4 ± 4.02	15.11 ± 5.61	26.8 ± 5.72	>4000[a]
10	BK-DCM	-	-	1.793 ± 5.00	1.497 ± 5.40	2.304 ± 5.96	6.771 ± 6.70	14.09 ± 5.68	26.35 ± 6.87	30.49 ± 5.64	49.32 ± 8.42	>4000[a]
11	BK-MeOH	10.29 ± 5.22	16.13 ± 3.92	26.42 ± 2.99	37.79 ± 4.60	56.85 ± 7.27	81.14 ± 6.06	88.55 ± 0.81	89.97 ± 0.53	-	-	94.24 ± 19.89
12	BK-H_2O	-	-	3.256 ± 4.36	5.436 ± 4.93	7.886 ± 6.37	11.27 ± 5.48	19.53 ± 5.53	32.34 ± 6.68	42.35 ± 7.06	71.69 ± 8.54	2,446 ± 527.4****

[a]The activity did not reach 50% at the highest tested concentration (4000 µg /ml), ****$p < 0.0001$ of samples against vitamin C ($n = 3$, ANOVA).

Table S2. Scavenging activity (%) and IC_{50} values of vitamin C from DPPH scavenging assay

No	Sample	DPPH scavenging percentage (%) at c the indicated concentration (µg/mL)							IC_{50} (µg/mL)
		1.56	3.125	6.25	12.5	25	50	100	
1	Vitamin C	1.10 ± 3.90	3.616 ± 2.89	5.15 ± 3.26	11.91 ± 4.52	24.59 ± 4.79	51.57 ± 9.65	86.88 ± 5.38	49.97 ± 10.76****

****$p < 0.0001$ was obtained from the comparison between vitamin C and FL-MeOH extract

Table S3. ABTS$^{\bullet+}$ scavenging activity of the extracts of *A. saligna*

No	Sample	ABTS scavenging percentage (%) at the indicated concentration (µg/mL)										IC_{50} (µg/mL)
		7.8125	15.625	31.25	62.5	125	250	500	1000	2000	4000	
1	FL-hex	-	-	3.576 ± 7.18	4.182 ± 6.08	4.969 ± 6.37	5.11 ± 8.65	8.021 ± 7.32	11.48 ± 3.99	18.64 ± 7.78	30.49 ± 4.99	>4000[a]
2	FL-DCM	-	-	2.534 ± 6.72	5.685 ± 6.17	10.12 ± 8.06	21.08 ± 6.79	29.53 ± 6.95	41.58 ± 6.71	54.65 ± 1.00	69.16 ± 0.85	1,579 ± 240.8****
3	FL-MeOH	7.884 ± 3.18	9.104 ± 2.21	12.09 ± 2.4	17.22 ± 1.371	27.71 ± 0.791	45.09 ± 0.597	62.72 ± 0.335	89.97 ± 2.278	-	-	316.6 ± 11.45****
4	FL-H_2O	-	-	2.719 ± 4.60	4.937 ± 5.69	6.898 ± 3.36	11.08 ± 3.37	18.01 ± 4.12	28.77 ± 3.38	43.6 ± 0.22	70.28 ± 5.39	2,433 ± 103.5****
5	LF-hex	-	-	-1.047 ± 1.45	2.395 ± 0.20	8.864 ± 0.42	7.013 ± 1.67	13.06 ± 1.39	23.57 ± 0.98	35.42 ± 1.41	65.73 ± 0.32	2,951 ± 75.9****
6	LF-DCM	-	-	3.193 ± 0.97	7.741 ± 3.71	12.18 ± 0.69	14.69 ± 0.60	23.84 ± 1.63	37.31 ± 0.13	56.49 ± 0.78	84.04 ± 0.18	1,633 ± 41.55****
7	LF-MeOH	9.964 ± 1.74	14.58 ± 0.85	20.71 ± 0.83	27.21 ± 0.03	43.98 ± 0.02	72.22 ± 0.55	99.63 ± 0.05	99.89 ± 0.05	-	-	146.7 ± 0.99
8	LF-H_2O	-	-	5.21 ± 0.17	6.959 ± 2.68	8.885 ± 1.94	18.07 ± 4.66	22.23 ± 6.35	31.56 ± 6.61	44.34 ± 0.39	71 ± 1.16	2,422 ± 148.8****
9	BK-hex	-	-	0.611 ± 1.77	1.13 ± 0.077	0.039 ± 0.64	3.449 ± 0.55	3.463 ± 2.23	9.726 ± 0.99	14.92 ± 3.10	30.56 ± 0.50	>4000[a]
10	BK-DCM	-	-	-3.323 ± 0.28	-2.756 ± 3.54	4.297 ± 1.63	13.23 ± 1.25	22.49 ± 0.33	30.96 ± 2.62	42.35 ± 1.82	62.81 ± 3.04	2,764 ± 165.3****
11	BK-MeOH	10.23 ± 6.29	16.81 ± 6.19	31.91 ± 5.20	52.46 ± 1.65	80.41 ± 7.44	96.87 ± 0.45	92.53 ± 3.37	99.87 ± 0.01	-	-	55.44 ± 6.84
12	BK-H_2O	-	-	-1.072 ± 6.05	3.195 ± 8.07	5.123 ± 3.34	11.29 ± 5.27	22.59 ± 4.59	40.95 ± 4.10	69.81 ± 1.83	93.68 ± 2.52	1,241 ± 97.93****

[a]The activity did not reach 50% at the highest tested concentration (4000 µg /mL), ****$p = 0.0001$ against vitamin C ($n = 3$, ANOVA).

Table S4. ABTS$^{\bullet+}$ scavenging activity (%) and IC_{50} values of vitamin C

No	Sample	ABTS scavenging percentage (%) at the indicated concentration (µg/mL)							IC_{50} (µg/mL)
		1.56	3.125	6.25	12.5	25	50	100	
1	Vitamin C	2.387 ± 5.29	1.026 ± 4.74	3.058 ± 5.04	6.223 ± 6.18	17.29 ± 3.44	31.21 ± 4.53	73.56 ± 2.64	72.25 ± 4.42****

****$p < 0.0001$ was summarised between vitamin C & FL-MeOH

Table S5a. Percentage of α-glucosidase inhibition (%) of flowers extracts

No	Extract	Percentage of inhibition (%) at the indicated concentration (µg/mL)						IC50 (µg/mL)
		3.125	6.25	12.5	25	50	100	
1	FL-hex	-2.523 ± 4.02	-3.158 ± 4.96	1.291 ± 0.72	2.861 ± 2.15	4.050 ± 1.79	4.652 ± 2.35	>100[a]
2	FL-DCM	-1.785 ± 2.53	-1.682 ± 2.22	0.339 ± 2.86	0.845 ± 3.09	0.277 ± 1.89	1.875 ± 5.5	>100[a]
3	FL-H_2O	-1.323 ± 2.81	-2.124 ± 3.21	-3.898 ± 4.64	-2.675 ± 5.69	-4.222 ± 6.54	-4.312 ± 6.65	>100[a]

[a]The activity did not reach 50% at the highest tested concentration (100 µg /mL).

Table S5b. Percentage of α-glucosidase inhibition (%) of methanolic flowers extract

No	Extract	Percentage of inhibition (%) at the indicated concentration (µg/mL)						IC50 (µg/mL)
		25	30	40	50	60	80	
1	FL-MeOH	20.04 ± 2.45	36.39 ± 3.26	62.55 ± 10.26	83.47 ± 0.86	85.59 ± 0.48	87.93 ± 0.43	34.93 ± 2.67***

***$p = 0.0004$ was from the inhibition of the extract *vs* acarbose ($n = 3$, ANOVA).

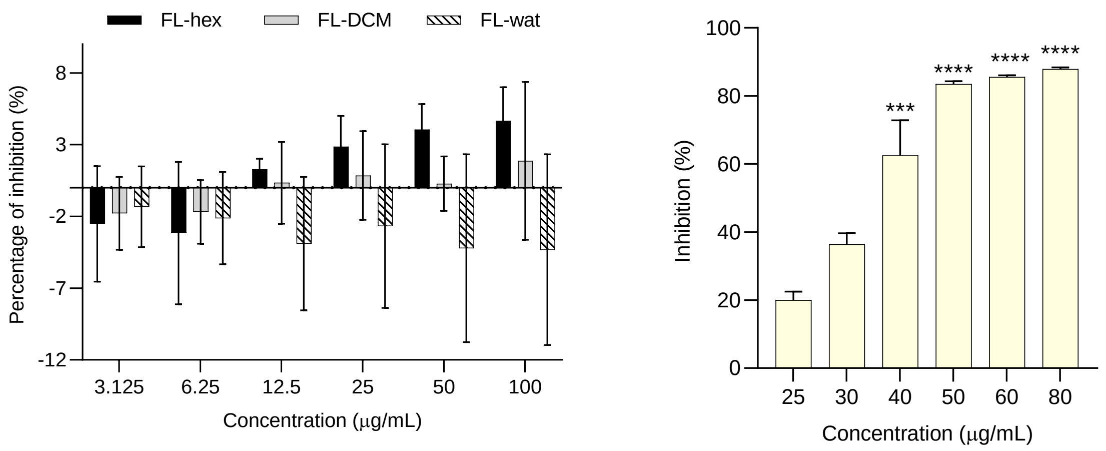

(a) **(b)**

Figure S2. Bar charts representing the dose-response relationship between the concentration of FL-hex, -DCM, and -wat extracts **(a)**; FL-MeOH **(b)** and inhibitory α-glucosidase percentage (***$p = 0.0003$; ****$p < 0.0001$ against inhibition at 25 µg/mL, $n = 3$, ANOVA)

Table S6a. Percentage of α-glucosidase inhibition (%) of leaves extracts

No	Extract	Percentage of inhibition (%) at the indicated concentration (µg/mL)						IC_{50} (µg/mL)
		31.25	62.5	125	250	500	1000	
1	LF-hex	-0.727 ± 3.28	-0.581 ± 3.69	17.89 ± 7.03	31.74 ± 16.58	50.91 ± 15.02	67.31 ± 11.81	285.5 ± 100.9
2	LF-DCM	-2.421 ± 6.73	0.188 ± 5.83	-2.172 ± 5.67	-342.1 ± 335	-340.6 ± 335.1	-8.070 ± 4.38	>1000[a]
3	LF-H_2O	-8.004 ± 8.82	-6.791 ± 8.11	-5.323 ± 7.40	-0.630 ± 4.68	22.97 ± 2.10	58.34 ± 3.61	882.6 ± 48.01

[a]The activity did not reach 50% at the highest tested concentration (1000 µg /ml), ANOVA, n = 3.

Table S6b. Percentage of α-glucosidase inhibition (%) of methanolic leaves extract

No	Extract	Percentage of inhibition (%) at the indicated concentration (µg/mL)						IC_{50} (µg/mL)
		25	30	40	50	60	80	
1	LF-MeOH	15.23 ± 2.68	28.86 ± 1.33	50.30 ± 2.33	80.31 ± 0.89	84.85 ± 0.95	86.79 ± 0.77	38.69 ± 1.01^{***}

$^{***}p = 0.0004$ was from the inhibition of the extract *vs* acarbose ($n = 3$, ANOVA).

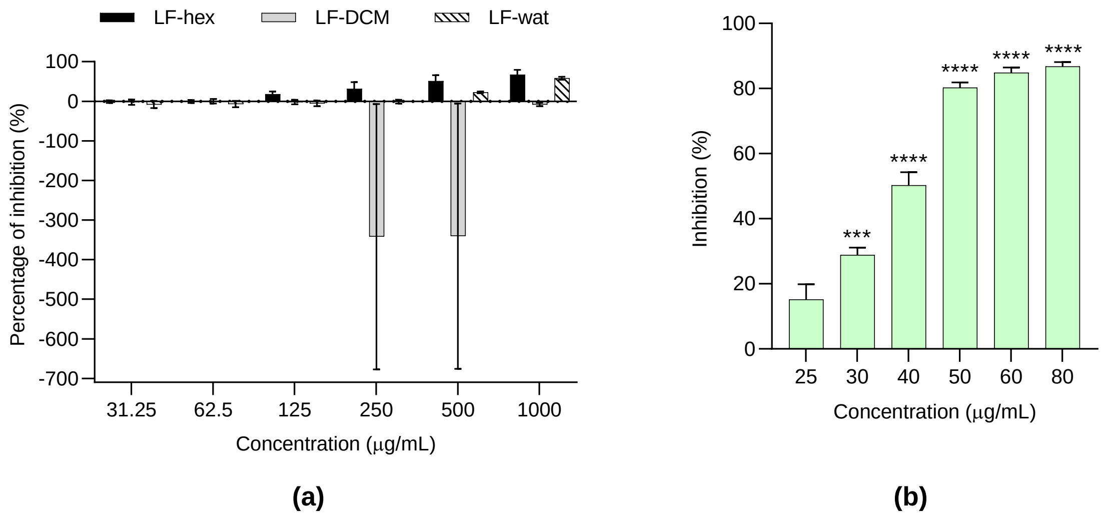

Figure S3. Bar charts representing the dose-response relationship between the concentration of LF-hex, -DCM, and -H_2O extracts **(a)**; LF-MeOH **(b)** and inhibitory α-glucosidase percentage ($^{***}p = 0.001$; $^{****}p < 0.0001$ against inhibition at 25 µg/mL, $n = 3$, ANOVA)

Table S7a. Percentage of α-glucosidase inhibition (%) of barks extracts

No	Extract	Percentage of inhibition (%) at the indicated concentration (μg/mL)						IC_{50} (μg/mL)
		**31.25**	**62.5**	**125**	**250**	**500**	**1000**	
1	BK-hex	1.114 ± 1.43	14.27 ± 6.99	17.77 ± 4.19	42.19 ± 5.31	77.07 ± 1.55	85.22 ± 2.05	289.9 ± 29.17
2	BK-DCM	-1.116 ± 2.49	-3.464 ± 1.02	4.055 ± 3.39	30.78 ± 13.83	53.23 ± 13.84	44.14 ± 16.84	>1000[a]
3	BK-H_2O	-4.953 ± 3.53	9.056 ± 3.14	22.09 ± 6.16	56.44 ± 7.51	79.68 ± 3.94	86.34 ± 0.09	23.27 ± 3.88****

[a]The activity did not reach 50% at the highest tested concentration (1000 μg /ml); $^{****}p = 0.0001$ was from the IC_{50} of BK-H_2O *vs* acarbose ($n = 3$, ANOVA).

Table S7b. Percentage of α-glucosidase inhibition (%) of methanolic barks extract

No	Extract	Percentage of inhibition (%) at the indicated concentration (μg/mL)						IC_{50} (μg/mL)
		**3**	**4**	**5**	**6**	**8**	**10**	
1	BK-MeOH	29.20 ± 4.85	40.10 ± 2.72	57.28 ± 7.44	75.46 ± 2.15	84.45 ± 0.17	85.35 ± 0.25	4.373 ± 0.24****

$^{****}p < 0.0001$ was from the IC_{50} of BK-MeOH *vs* acarbose ($n = 3$, ANOVA).

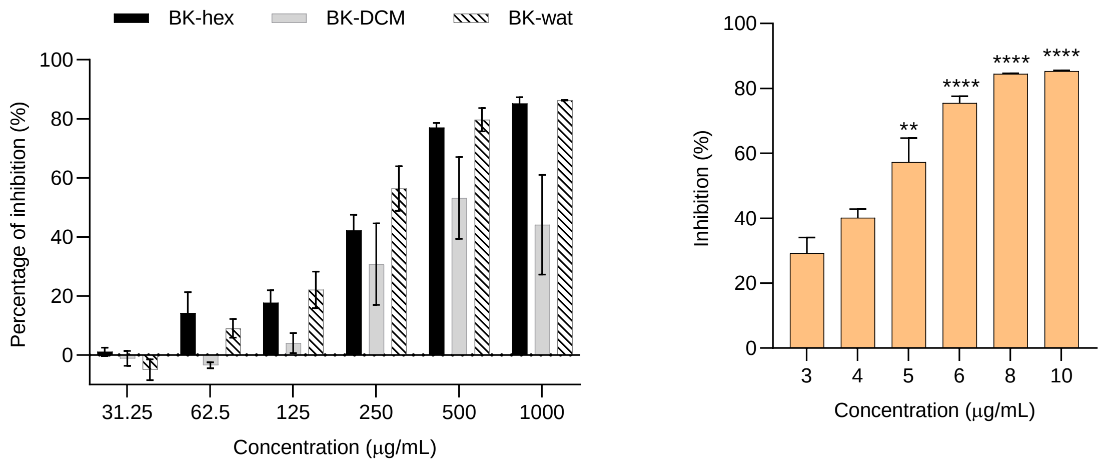

(a) **(b)**

Figure S4. Bar charts representing the dose-response relationship between the concentration of BK-hex, -DCM, and -wat extracts **(a)**; BK-MeOH **(b)** and inhibitory α-glucosidase percentage ($^{**}p = 0.003$; $^{****}p < 0.0001$ against inhibition at 3 μg/mL, $n = 3$, ANOVA)

Table S8. Percentage of α-glucosidase inhibition (%) of acarbose

No	Sample	Percentage of inhibition (%) at the indicated concentration (µg/mL)						IC_{50} (µg/mL)
		31.25	62.5	125	250	500	1000	
1	Acarbose	16.62 ± 3.95	23.06 ± 3.99	35.20 ± 2.41	47.16 ± 2.18	59.39 ± 1.37	70.64 ± 1.38	254 ± 22.18

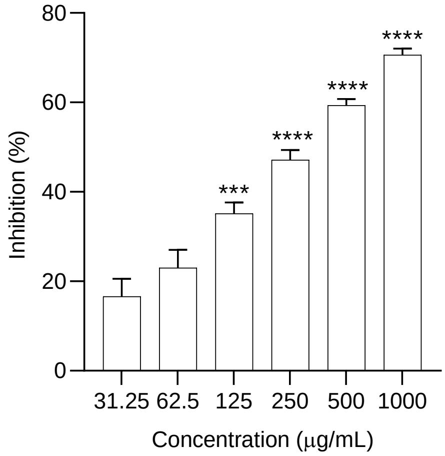

Figure S5. Bar charts representing the dose-response relationship between acarbose concentration and inhibitory α-glucosidase percentage ($^{***}p = 0.0006$; $^{****}p < 0.0001$ against inhibition at 31.25 µg/mL, $n = 3$, ANOVA)

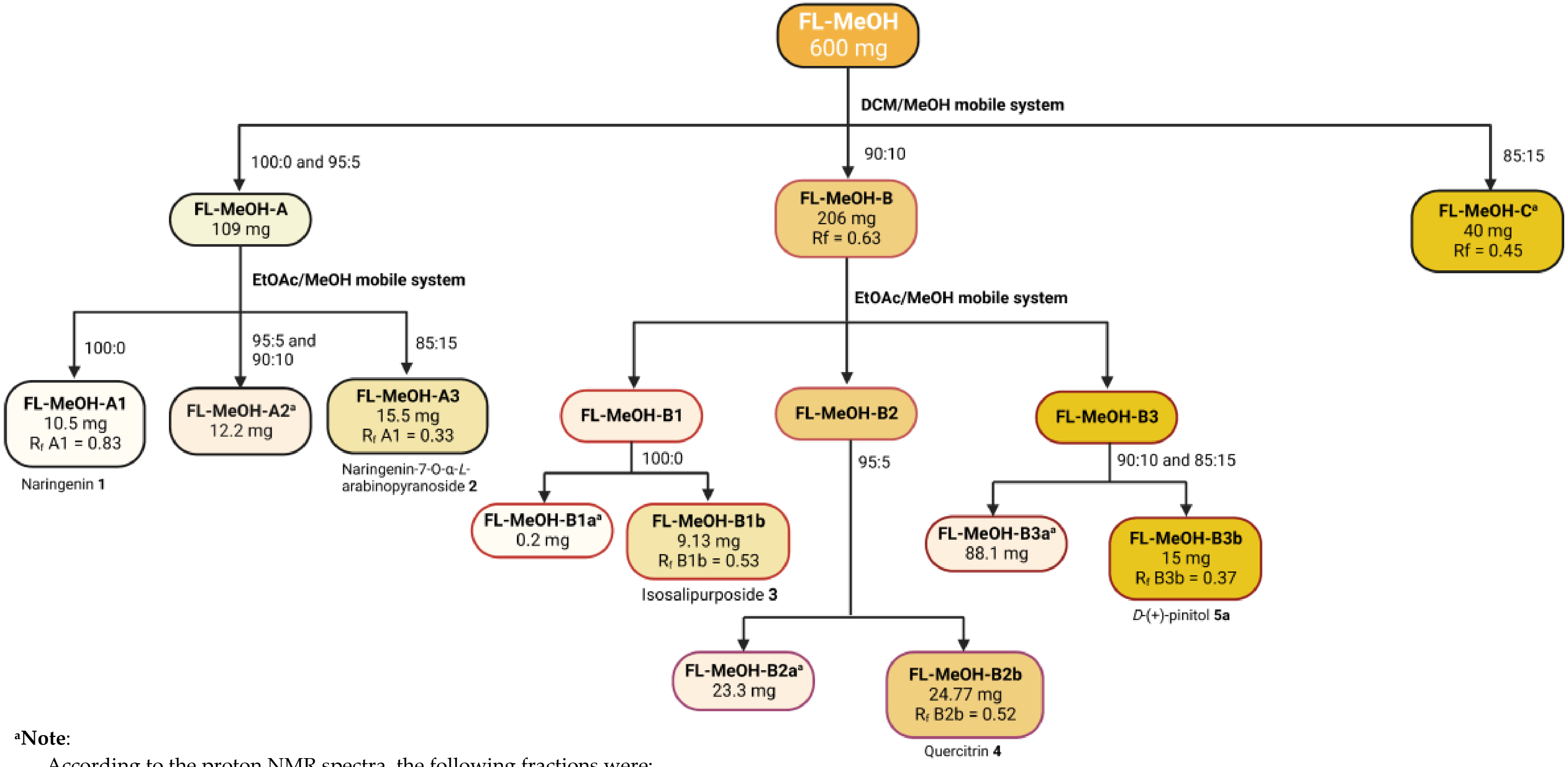

[a]**Note**:

According to the proton NMR spectra, the following fractions were:

1) FL-MeOH-A2 = mixture of subfraction A1 and A3
2) FL-MeOH-B1a = mixture of hydrocarbons (δ ^{1}H = 0–1.8 ppm)
3) FL-MeOH-B2a = impure subfraction B2b
4) FL-MeOH-B3a = impure subfraction B3b
5) FL-MeOH-C = impure fraction B

Figure S6. Schematic representation of the outcomes of fractionation of FL-MeOH extract of *A. saligna*

Table S9. ^{1}H NMR (400 MHz, CD_3OD) and ^{13}C NMR (100 MHz, CD_3OD) of isolate FL-MeOH-A1 compared to reported naringenin **1**

Atoms	FL-MeOH-A1		Naringenin 1[a]	
	**δ ^{1}H in ppm (m, *J* Hz, integration)**	**δ ^{13}C (ppm)**	**δ ^{1}H in ppm (m, *J* Hz, integration)**	**δ ^{13}C (ppm)**
2	5.26 (dd; 12.92, 2.9; 1H)	80.63	5.34 (dd; 13, 3; 1H)	80.5
3a	3.03 (dd; 17.12, 12.92; 1H)	44.2	3.1 (dd; 17, 13; 1H)	44
3b	2.62 (dd; 17.04, 3.06; 1H)		2.7 (dd; 17, 3; 1H)	
4	-	197.93	-	197.8
5	-	165.62	-	165.5
6	5.81 (d; 2.16; 1H)	97.19	5.88 (d; 2; 1H)	97.1
7	-	168.5	-	168.4
8	5.82 (d; 2.16; 1H)	96.31	5.9 (d; 2; 1H)	96.2
9	-	165.04	-	164.9
10	-	103.5	-	103.4
1′	-	131.22	-	131.1
2′	7.23 (dd; 6.76, 1.78; 1H)	129.18	7.31 (m; 1H)	129
3′	6.74 (dd; 6.64, 2.02; 1H)	116.47	6.82 (m; 1H)	116.4
4′	-	159.18	-	159
5′	6.74 (dd; 6.64, 2.02; 1H)	116.47	6.82 (m; 1H)	116.4
6′	7.23 (dd; 6.76, 1.78; 1H)	129.18	7.31 (m; 1H)	129

[a]Du, Q., Jerz, G.; Winterhalter, P., Preparation of three flavonoids from the bark of Salix alba by high-speed countercurrent chromatographic separation. *Journal of liquid chromatography & related technologies* **2004,** *27*, 3257-3264.

Table S10. ^{1}H NMR (400 MHz, CD_3OD) and ^{13}C NMR (100 MHz, CD_3OD) of FL-MeOH-B1b compared to reported isosalipurposide **3**

Atoms	FL-MeOH-B1b		Isosalipurposide 3[a]	
	**δ ^{1}H in ppm (m, *J* Hz, integration)**	**δ ^{13}C (ppm)**	**δ ^{1}H in ppm (m, *J* Hz, integration)**	**δ ^{13}C (ppm)**
**1**	-	128.47	-	128.3
2	7.61 (d; 8.64; 1H)	131.74	7.62 (d; 8.0; 1H)	131.8
3	6.85 (d; 8.72; 1H)	116.85	6.87 (d; 8.0; 1H))	116.9
4	-	161.01	-	161.8
5	6.85 (d; 8.72; 1H)	116.85	6.87 (d; 8.0; 1H)	132.2
6	7.61 (d; 8.64; 1H)	131.74	7.62 (d; 8.0; 1H)	116.9
α	8.01 (d; 15.52; 1H)	125.88	8.02 (d; 15.0; 1H)	144.2
β	7.69 (d; 15.56; 1H)	144.12	7.68 (d; 15.0; 1H)	125.8
C=O	-	194.44	-	194.8
1′	-	107.46	-	107.8
2′	-	165.77	-	165.9
3′	6.18 (d; 2.24; 1H)	95.63	6.26 (s; 1H)	95.9
4′	-	161.75	-	161.1
5′	6.02 (d; 2.28; 1H)	98.35	6.03 (s; 1H)	98.5
6′	-	167.70	-	161.4
1″	5.09 (d; 7.4; 1H)	101.82	5.15 (d; 7.8; 1H)	101.9
2″	3.44 (t; 8.75; 1H)	74.97	3.39–3.46 (m; 1H)	75.3
3″	3.37 (t; 8.25; 1H)	78.45	3.60 (dd; 10.5, 7.5; 1H)	76.3

Atoms	FL-MeOH-B1b		Isosalipurposide 3[a]	
	δ ^{1}H in ppm (m, *J* Hz, integration)	δ ^{13}C (ppm)	δ ^{1}H in ppm (m, *J* Hz, integration)	δ ^{13}C (ppm)
4″	3.47 (d; 7.5; 1H)	71.10	3.47–3.50 (m; 1H)	72.1
5″	3.39 (m; 1H)	78.41	3.54–3.57 (m; 1H)	76.7
6″ a	3.47 (dd; 12, 5.32; 1H)	62.34	3.76 (dd; 12.6, 1.5; 1H)	62.3
**6″ b**	3.99 (dd; 11.36, 5.28; 1H)	62.34	3.94 (dd; 12.6, 1.5; 1H)	62.3

[a]Hendra, R., Willis, A.; Keller, P.A., Phytochemical studies on the Australian native plant species *Acacia pycnantha* and *Jacaranda mimosifolia* D. Don.. *Natural product research* **2019,** *33*, 1997-2003

Table S11. ^{1}H NMR (400 MHz, CD_3OD) and ^{13}C NMR (100 MHz, CD_3OD) of FL-MeOH-B2b compared to reported quercitrin **4**

Atoms	FL-MeOH-B2b		Quercitrin 4[a]	
	δ ^{1}H ppm (m, *J* Hz, integration)	δ ^{13}C (ppm)	δ ^{1}H ppm (m, *J* Hz, integration)	δ ^{13}C (ppm)
**2**	-	146.57	-	149.9
**3**	-	136.38	-	136.2
**4**	-	179.80	-	179.6
**5**	-	158.68	-	163.2
**6**	6.21 (d; 2.12; 1H)	99.95	6.13 (d; 2.5; 1H)	100.2
**7**	-	166.01	-	167.2
**8**	6.38 (d; 2.08; 1H)	94.85	6.29 (d; 2.5; 1H)	95.3
**9**	-	163.37	-	158.6
**10**	-	106.05	-	105.6
**1′**	-	123.12	-	123.1
**2′**	7.35 (s; 1H)	117.07	7.28 (s; 1H)	116.9
**3′**	-	149.95	-	146.4
**4′**	-	159.46	-	159.2
**5′**	6.92 (d; 8.28; 1H)	116.51	6.86 (d; 7.9; 1H)	116.4
**6′**	7.32 (dd; 8.28, 2.14; 1H)	123.01	7.25 (d; 7.9; 1H)	122.8
**1″**	5.36 (d; 1.48; 1H)	103.69	5.29 (d; 1.2; 1H)	103.5
**2″**	4.23 (dd; 3.28, 1.68; 1H)	72.05	4.17 (m; 1H)	71.9
**3″**	3.76 (dd; 9.36, 3.44; 1H)	72.26	3.70 (d; 6.7; 1H)	72.2
**4″**	3.36 (t; 9.44; 1H)	73.40	3.32 (d; 9.6; 1H)	73.4
**5″**	3.42 (d; 6.12; 1H)	72.18	3.35 (m; 1H)	72
**6″**	0.95 (d; 6.08; 3H)	17.80	0.86 (d; 6.1; 3H)	17.7

[a]Kim, Y.-K., et al., Isolation of flavonol rhamnosides from *Loranthus tanakae* and cytotoxic effect of them on human tumor cell lines. *Archives of pharmacal research* **2004,** *27*, 44-47.

Table S12. ^{1}H NMR (400 MHz, D_2O) and ^{13}C NMR (100 MHz, D_2O) of isolate FL-MeOH-B3b compared to reported 3-*O*-methyl-*D*-chiro-inositol (*D*-pinitol) **5a**

**Atoms**	**FL-MeOH-B3b**		***D*-pinitol 5a**[a,b]	
	**δ ^{1}H in ppm (m, *J* Hz, integration)**	**δ ^{13}C (ppm)**	**δ ^{1}H in ppm (m, *J* Hz, integration)**	**δ ^{13}C (ppm)**
**1,6**	3.87 (m; 6.08, 2.08; 2H)	71.62, 71.42	3.85 (m; 2H)	71.89, 71.67
**2**	3.68 (dd; 9.96, 2.86; 1H)	69.78	3.66 (dd; 9.90, 2.60; 1H)	70.02
**3**	3.21 (t; 9.64; 1H)	82.73	3.19 (t; 9.72; 1H)	82.96
**4**	3.51 (t; 9.56; 1H)	72.08	3.50 (t; 9.76; 1H)	72.32
**5**	3.62 (dd; 9.96, 2.86; 1H)	70.49	3.61 (dd; 9.98, 2.60; 1H)	70.73
**7**	3.63 (s; 3H)	59.68	3.45 (s; 3H)	59.88

[a]Raya-Gonzalez, D., et al., D-(+)-pinitol, a component of the heartwood of *Enterolobium cyclocarpum* (Jacq.). *Griseb. Zeitschrift für Naturforschung* 2008, *63*, 922-924.

[b]Anderson, A. B., MacDonald, D.; Fischer, H.O., The structure of pinitol. *J. Am. Chem. Soc.* 1952, *74*, 1479-1480

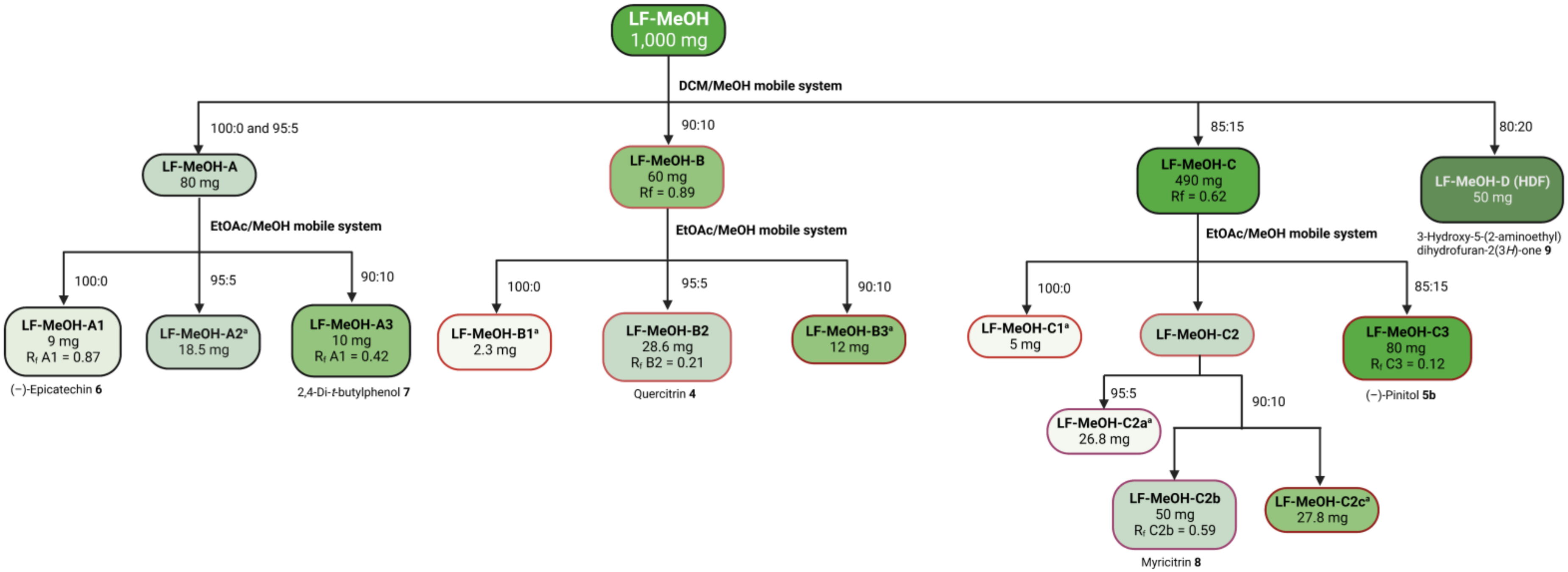

[a]**Note**:

According to the proton NMR spectra, the following fractions were:

1) LF-MeOH-A2 = mixture of subfraction A1 and A3
2) LF-MeOH-B1 = mixture of hydrocarbons (δ ^{1}H = 0–1.8 ppm)
3) LF-MeOH-B3 = impure subfraction B2
4) LF-MeOH-C1 = mixture of hydrocarbons (δ ^{1}H = 0–1.8 ppm)
5) LF-MeOH-C2a = impure subfraction C2b
6) LF-MeOH-C2c = mixture of subfraction C2b and C3

Figure S7. Schematic representation of the outcomes of fractionation of LF-MeOH extract of *A. saligna*

Table S13. ^{1}H NMR (400 MHz) and ^{13}C NMR (100 MHz) of epicatechin in CD_3OD isolated from LF-MeOH-A1 extract compared to reference (–)-epicatechin **6**

Atoms	LF-MeOH-A1		(–)-Epicatechin 6[a]	
	**δ ^{1}H in ppm (m, *J* in Hz, integration)**	**δ ^{13}C (ppm)**	**δ ^{1}H in ppm (m, *J* in Hz, integration)**	**δ ^{13}C (ppm)**
**2**	4.58 (d; 7.52; 1H)	81.47	4.82 (br s; 1H)	79.88
**3**	3.99 (m; 1H)	67.42	4.19 (m; 1H)	67.49
**4a**	2.52 (dd; 16.08, 8.2; 1H)	27.13	2.73 (dd; 16.8, 2.9; 1H)	29.26
**4b**	2.87 (dd; 16.12, 5.4; 1H)		2.87 (dd; 16.8, 4.5; 1H)	
**5**	-	156.40	-	158
**6**	5.95 (d; 2.2; 1H)	94.88	5.94 (d; 2.3; 1H)	96.38
**7**	-	156.45	-	157.67
**8**	5.87 (d; 2.2; 1H)	94.1	5.97 (d; 2.3; 1H)	95.88
**9**	-	155.53	-	157.37
**10**	-	99.41	-	100.06
**1′**	-	130.82	-	132.28
**2′**	6.86 (d; 1.6; 1H)	113.86	6.98 (d; 1.9; 1H)	115.32
**3′**	-	144.84	-	145.78
**4′**	-	144.86	-	145.95
**5′**	6.78 (d; 8.08; 1H)	114.67.	6.76 (d; 8.4; 1H)	115.88
**6′**	6.74 (dd; 8.16, 1.88; 1H)	118.64	6.81 (dd; 8.4, 1.9; 1H)	119.39

[a]Kim, H. J., et al., A new epicatechin gallate and calpain inhibitory activity from *Orostachys japonicus*. *Fitoterapia* **2009,** *80*, 73-76.

Table S14. ^{1}H NMR (400 MHz) and ^{13}C NMR (100 MHz) of LF-MeOH-A3 in CD_3OD compared to 2,4-di-*t*-buylphenol **47**

Atoms	LF-MeOH-A3		2,4-di-*t*-buylphenol 7[a]	
	**δ ^{1}H in ppm (m, *J* Hz, integration)**	**δ ^{13}C (ppm)**	**δ ^{1}H in ppm (m, *J* Hz, integration)**	**δ ^{13}C (ppm)**
**1**	-	154.93	-	153.38
**2**	-	136.27	-	134.77
**3**	7.24 (d; 2.24; 1H)	124.45	7.24 (d; 2.4; 1H)	122.91
**4**	-	142.45	-	140.96
**5**	7.02 (dd; 8.32, 2.48; 1H)	124.37	7.02 (dd; 8.2, 2.4; 1H)	122.82
**6**	6.640 (d; 8.32; 1H)	116.73	6.64 (d; 8.2; 1H)	115.19
**7**	-	35.85	-	34.31
**8**	-	35.12	-	33.58
**9**	1.4 (s; 9H)	30.23	1.40 (s, 9H)	28.67
**10**	1.29 (s; 9H)	32.29	1.29 (s, 9H)	30.76

[a]Belghit, S., et al., Activity of 2, 4-Di-tert-butylphenol produced by a strain of *Streptomyces mutabilis* isolated from a Saharan soil against *Candida albicans* and other pathogenic fungi. *Journal de mycologie medicale* **2016,** *26*, 160-169.

Table S15. ^1H NMR (400 MHz, CD_3OD) and ^{13}C NMR (100 MHz, CD_3OD) of isolate LF-MeOH-C2b compared to reported myricetin–3–*O*–α–L–rhamnopyranoside (Myricitrin) **8**

**Atoms**	**LF-MeOH-C2b**		**Myricitrin 8**[a]	
	**δ ¹H ppm (m, *J* Hz, integration)**	**δ ¹³C (ppm)**	**δ ¹H ppm (m, *J* Hz, integration)**	**δ ¹³C (ppm)**
**2**	-	159.59	-	159.2
**3**	-	136.46	-	136.1
**4**	-	179.83	-	179.5
**5**	-	163.38	-	163.1
**6**	6.21 (d; 2; 1H)	99.96	6.19 (d; 1.8; 1H)	99.7
**7**	-	166.12	-	164.0
**8**	6.37 (d; 2; 1H)	94.83	6.35 (d; 2.3; 1H)	94.6
**9**	-	158.61	-	158.4
**10**	-	106.00	-	105.6
**1′**	-	122.06	-	121.7
**2′**	6.96 (s; 2H)	109.69	6.94 (s; 2H)	109.6
**3′**	-	147.02	-	146.7
**4′**	-	137.99	-	137.7
**5′**	-	147.02	-	146.7
**6′**	6.96 (s; 2H)	109.69	6.94 (s; 2H)	109.6
**1″**	5.33 (s; 1H)	103.79	5.30 (d; 1.8; 1H)	103.5
**2″**	4.24 (s; 1H)	72.04	4.21 (dd; 3.2, 1.8; 1H)	71.7
**3″**	3.8 (dd; 9.4, 3.48; 1H)	72.27	3.76–3.78 (dd; 9.4, 3.4; 1H)	72.0
**4″**	3.37 (t; 9.52; 1H)	73.50	3.31–3.34 (m; 1H)	73.2
**5″**	3.53 (dd; 9.66, 6.1; 1H)	72.19	3.48–3.54 (m; 1H)	71.9
**6″**	0.97 (d; 6.16; 3H)	17.82	0.94–0.96 (m; 3H)	17.5

[a]Hwang, I. W., Chung, S. K., Isolation and identification of myricitrin, an antioxidant flavonoid, from *Daebong persimmon* peel. *Preventive nutrition and food science* **2018,** *23*, 341.

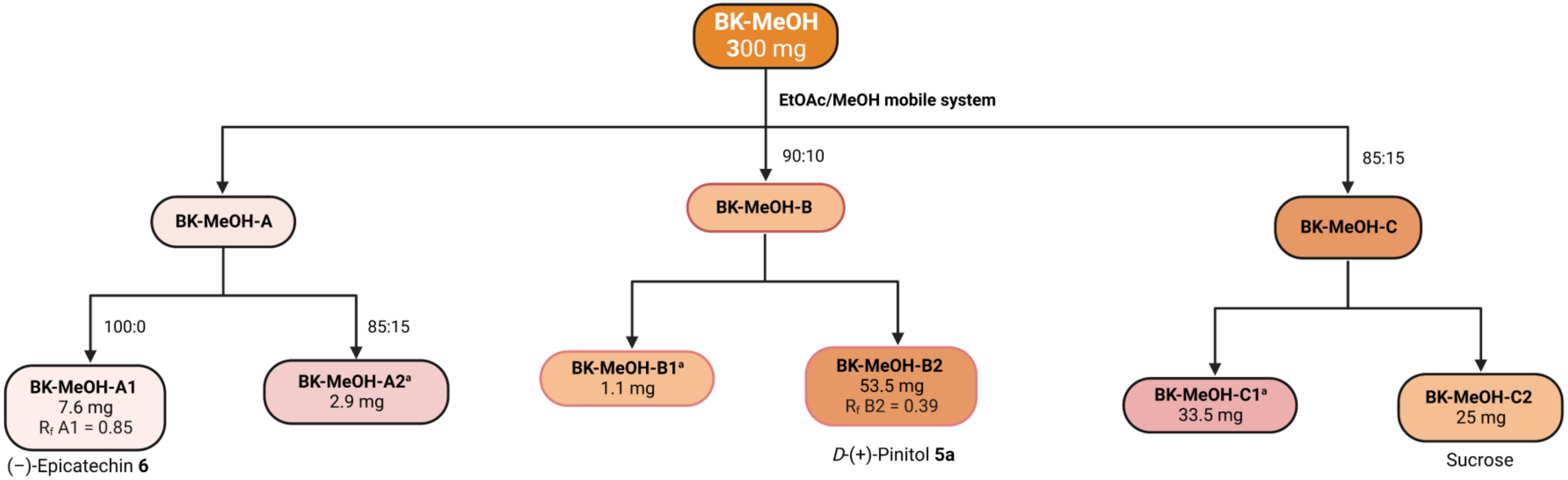

[a]**Note**:

According to the proton NMR spectra, the following fractions were:

1) BK-MeOH-A2 = impure fraction A1

2) BK-MeOH-B1 = impure fraction B1

3) BK-MeOH-C1 = mixture of B2 and C2

Figure S8. Schematic representation of the outcomes of fractionation of BK-MeOH extract of *A. saligna*

Table S16. ^1H NMR (400 MHz, D_2O) and ^{13}C NMR (100 MHz, D_2O) of BK-MeOH-C compared to reported sucrose

**Atoms**	**BK-MeOH-C**		**Sucrose**[a]	
	**δ ^{1}H in ppm (m, *J* Hz, integration)**	**δ ^{13}C (ppm)**	**δ ^{1}H in ppm (m, *J* Hz, integration)**	**δ ^{13}C (ppm)**
**1**	5.44 (d; 3.84; 1H)	92.74	5.38 (d; 3.88; 1H)	94.66
**2**	3.58 (dd; 10, 3.84; 1H)	71.63	3.52 (dd; 10, 3.84; 1H)	73.55
**3**	3.79 (t; 9.4; 1H)	73.12	3.72 (t; 9.56; 1H)	75.05
**4**	3.49 (t; 9.46; 1H)	69.78	3.43 (t; 9.42; 1H)	71.70
**5**	3.89 (m; 1H)	72.96	3.83 (m; 1H)	74.88
**6**	3.85 (d; 2; 2H)	62.93	3.78 (d; 2.96; 2H)	62.59
**1′**	3.70 (s; 2H)	61.91	3.63 (s; 2H)	63.82
**2′**	-	104.24	-	106.17
**3′**	4.24 (d; 8.76; 1H)	76.98	4.18 (d; 8.76; 1H)	78.88
**4′**	4.00 (t; 8.54; 1H)	74.55	4.01 (t; 1H; 8.56)	76.47
**5′**	3.93 (m; 1H)	81.92	3.86 (m; 1H)	83.85
**6′**	3.84 (s; 2H)	60.68	3.79 (s; 2H)	64.84

[a] Hernández-García, E., et al., Nuclear magnetic resonance spectroscopy data of isolated compounds from *Acacia farnesiana* (L) Willd fruits and two esterified derivatives. *Data in brief*, **2019**, *22*, 255-268.

Table S17. The DPPH scavenging properties of the isolated compounds

No	Sample	**Scavenging percentage (%) at** the indicated concentration **(µM)**								IC_{50} (µM)
		**62.5**	**125**	**250**	**500**	**1,000**	**2,000**	**5,000**	**10,000**	
1	Isosalipurposide **3**	-	15.14 ± 1.24	18.65 ± 1.74	25.34 ± 0.46	37.83 ± 2.00	56.78 ± 2.75	61.44 ± 2.74	-	1,559 ± 28.16***
2	Naringenin **1**	-	-	10.06 ± 1.61	12.34 ± 1.02	14.91 ± 0.56	21.46 ± 0.74	26.55 ± 3.29	34.51 ± 0.39	>10,000[a]
3	Quercitrin **4**	9.75 ± 0.72	18.71 ± 1.82	38.91 ± 2.00	68.99 ± 1.13	86.6 ± 0.39	90.5 ± 0.17	-	-	322.6 ± 14.05****
4	Myricitrin **8**	14.44 ± 1.88	28.65 ± 0.70	57.71 ± 1.33	93.77 ± 0.50	95.01 ± 0.14	95.38 ± 0.17	-	-	199.9 ± 4.83****
5	Naringenin-7-O-α-L-arabinopyranose **2**	-	-	1.62 ± 0.37	2.62 ± 1.68	3.98 ± 0.47	7.35 ± 2.14	16.08 ± 5.11	19.62 ± 0.53	>10,000[a]
6	*D*-(+)-pinitol **5a**	-	-	8.23 ± 2.52	15.77 ± 2.41	29.25 ± 3.11	58.31 ± 1.55	88.25 ± 1.58	87.38 ± 0.56	1,675 ± 65.72****
7	(–)-pinitol **5b**	-	-	12.39 ± 3.61	14.62 ± 1.37	20.22 ± 0.16	28.10 ± 0.57	42.58 ± 1.00	59.93 ± 2.21	6,865 ± 69.08****
8	(–)-Epicatechin **6**	14.04 ± 3.44	23.44 ± 2.00	43.22 ± 1.91	65.65 ± 9.73	87.84 ± 6.31	95.28 ± 0.28	-	-	278 ± 8.62****
9	2,4-Di-*t*-butylphenol **7**	-	-	8.00 ± 3.65	10.16 ± 3.02	12.05 ± 3.42	21.75 ± 4.18	24.47 ± 3.73	36.9 ± 4.20	>10,000[a]
10	3-Hydroxy-5-(2-aminoethyl) dihydrofuran-2(3*H*)-one **9**	-	-	-6.10	-3.31	0.02 ± 5.07	5.78 ± 5.61	8.92 ± 4.58	14.37 ± 5.29	>10,000[a]
11	Vitamin C	1.86 ± 1.45	5.71 ± 2.28	6.738 ± 1.43	20.65 ± 1.62	46.58 ± 2.18	87.17 ± 2.15	-	-	1,072 ± 47.64

[a]The activity did not reach 50% at the highest tested concentration (10 mM); ***$p = 0.0002$, ****$p < 0.0001$ were from the IC_{50} of the compound *vs* vitamin C ($n = 3$, ANOVA, Tukey).

Table S18. The ABTS scavenging properties of the isolated compounds

No	Sample	Scavenging percentage (%) at the indicated concentration (μM)								IC_{50} (μM)
		62.5	125	250	500	1,000	2,000	5,000	10,000	
1	Isosalipurposide **3**	-	-	13.98 ± 0.26	23.37 ± 1.96	36.21 ± 2.37	56.01 ± 2.29	80.17 ± 1.90	93.27 ± 0.45	1,686 ± 95.26****
2	Naringenin **1**	-	19.55 ± 1.05	26.91 ± 0.12	35.88 ± 0.80	43.71 ± 0.72	55.46 ± 4.06	79.89 ± 0.76	-	1,525 ± 316.5****
3	Quercitrin **4**	14.01 ± 2.14	24.82 ± 0.34	37.36 ± 0.73	63.06 ± 1.37	85.56 ± 1.67	99.03 ± 0.06	-	-	355.3 ± 12.08
4	Myricitrin **8**	14.29 ± 4.22	29.41 ± 2.13	44.56 ± 1.02	67.66 ± 1.4	82.1 ± 1.07	91.98 ± 0.95	-	-	285.9 ± 7.21
5	Naringenin-7O-*α*-L-arabinopyranose **2**	-	15.36 ± 2.00	24.98 ± 1.42	29.92 ± 1.69	34.82 ± 1.07	38.65 ± 3.00	56.29 ± 1.34	-	4,146 ± 99.15****
6	*D*-(+)-pinitol **5a**	6.55 ± 5.32	15.61 ± 5.01	27.6 ± 4.14	50.66 ± 2.31	92.43 ± 0.19	99.96 ± 0.34	-	-	475 ± 24.20
7	(–)-pinitol **5b**	-	5.87 ± 0.16	2.98 ± 0.83	18.5 ± 2.05	28.61 ± 1.83	48.48 ± 1.37	82.87 ± 3.41	-	2,096 ± 70.40****
8	(–)-Epicatechin **6**	35.88 ± 3.58	63.17 ± 4.09	90.85 ± 2.43	99.56 ± 0.04	99.67 ± 0.09	99.85 ± 0.13	-	-	92.58 ± 13.03
9	2,4-Di-*t*-butylphenol **7**	-	10.89 ± 1.68	11.94 ± 2.44	19.29 ± 0.04	27.88 ± 1.19	41.91 ± 0.25	70.9 ± 3.48	-	2,715 ± 64.02****
10	3-Hydroxy-5-(2-aminoethyl) dihydrofuran-2(3*H*)-one **9**	-	4.35 ± 4.02	8.67 ± 6.85	1.68 ± 2.58	5.84 ± 1.27	10.46 ± 3.85	22.49 ± 5.44	-	>10,000[a]
11	Vitamin C	9.40 ± 1.74	13.86 ± 3.70	30.4 ± 6.61	53.52 ± 4.17	80.4 ± 1.65	96.39 ± 1.71	-	-	460.2 ± 56.29

[a]The activity did not reach 50% at the highest tested concentration (5 mM). ****$p < 0.0001$ was from the IC_{50} of the compound *vs* vitamin C ($n = 3$, ANOVA, Tukey).

Table S19. GC analysis of BK-MeOH

No	Group	Compound	Molecular formula	Structure
1	Unsaturated carboxylic acid	*trans*-Cinnamic acid	$C_9H_8O_2$	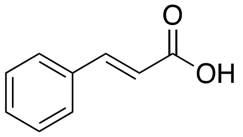
2	Polyols	4-C-Methyl-*myo*-inositol (lamitol)	$C_7H_{14}O_6$	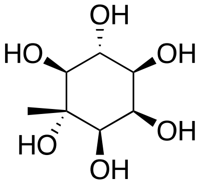
3	Amino acid (primary metabolites)	*D*-Asparagine	$C_4H_8N_2O_3$	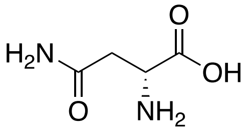
4	Nucleoside	Thymidine-5′-monophosphate	$C_{10}H_{15}N_2O_8P$	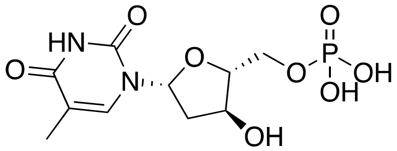

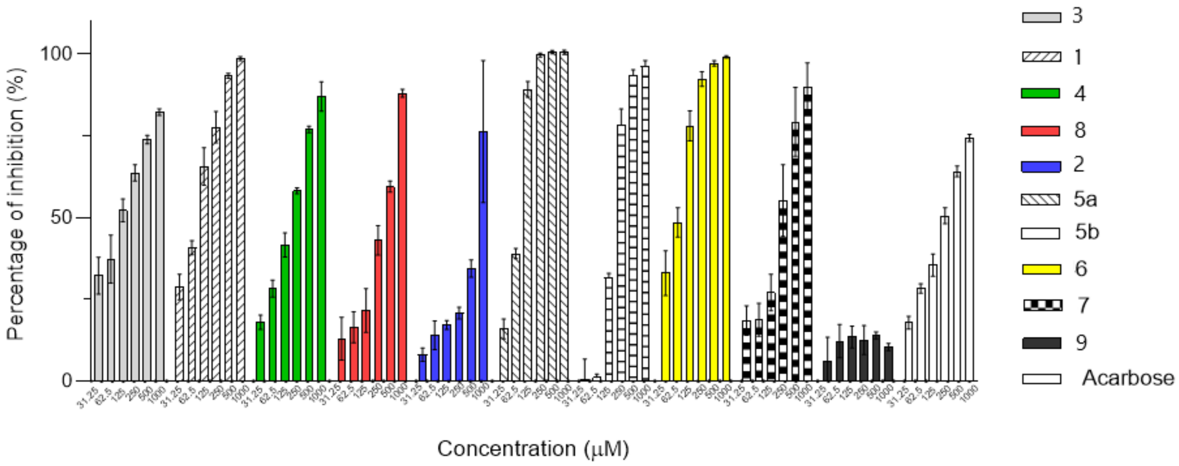

Fig S9. Bar graphs representing the inhibitory activity (%) of isolated compounds against the α-glucosidase enzyme.

Compounds. **3**: isosalipurposide; **1**: naringenin; **4**: quercitrin; **8**: myricitrin; **2**: naringenin-7O-α-L-arabinopyranose; **5a**: *D*-(+)-pinitol, **5b**: (–)-pinitol; **6**: (–)-epicatechin; **7**: 2,4-di-*t*-butylphenol; **9**: 3-hydroxy-5-(2-aminoethyl) dihydrofuran-2(3*H*)-one**.**

Table S20. The quantified inhibitory activity of the isolated compounds against the α-glucosidase enzyme

No	Sample	Inhibitory percentage (%) at the corresponding concentration (μM)						IC_{50} (μM)
		**31.25**	**62.5**	**125**	**250**	**500**	**1,000**	
1	Isosalipurposide **3**	32.27 ± 5.61	37.36 ± 7.32	52.19 ± 3.49	63.69 ± 2.47	73.88 ± 1.29	82.3 ± 1.00	116.5 ± 26.40
2	Naringenin **1**	28.81 ± 3.89	40.78 ± 2.19	65.64 ± 5.77	77.64 ± 4.81	93.37 ± 0.75	98.59 ± 0.61	89.71 ± 10.22*
3	Quercitrin **4**	18.01 ± 2.18	28.32 ± 2.61	41.66 ± 3.69	58.19 ± 0.84	76.97 ± 0.99	87.03 ± 4.46	177.3 ± 11.34
4	Myricitrin **8**	13.01 ± 6.59	16.52 ± 4.71	21.58 ± 6.71	43.06 ± 4.48	59.48 ± 1.73	88.06 ± 1.19	351.6 ± 24.88
5	Naringenin-7O-α-L-arabinopyranose **2**	8.11 ± 2.03	14.07 ± 4.44	17.2 ± 1.41	20.81 ± 1.80	34.41 ± 2.66	76.34 ± 21.72	769.1 ± 95.82****
6	*D*-(+)-pinitol **5a**	16 ± 3.03	39 ± 1.59	89.22 ± 2.40	99.74 ± 0.57	100.6 ± 0.54	100.5 ± 0.64	74.69 ± 0.23*
7	(–)-pinitol **5b**	0.29 ± 6.45	1.18 ± 0.97	31.64 ± 1.35	78.43 ± 4.73	93.46 ± 1.77	96.17 ± 1.85	164.2 ± 8.36
8	(–)-Epicatechin **6**	33.01 ± 6.84	48.53 ± 4.50	78.04 ± 4.6	92.36 ± 2.23	97.14 ± 0.82	99.15 ± 0.38	63.58 ± 11.83*
9	2,4-Di-*t*-butylphenol **7**	18.57 ± 4.45	18.73 ± 5.06	27.11 ± 5.58	55.17 ± 10.98	79.26 ± 10.49	89.86 ± 7.42	259 ± 58.34
10	3-Hydroxy-5-(2-aminoethyl) dihydrofuran-2(3*H*)-one **9**	5.99 ± 7.41	12.28 ± 5.13	13.5 ± 3.35	12.55 ± 4.46	14.01 ± 1.03	10.47 ± 1.06	>1000[a]
11	Acarbose	17.88 ± 1.97	28.34 ± 1.44	35.48 ± 3.47	50.6 ± 2.46	64.13 ± 1.65	74.34 ± 1.09	239.9 ± 31.74

[a]The activity did not reach 50% at the highest tested concentration (1 mM); $^{*}p = 0.03$; $^{****}p < 0.0001$ were from the inhibition of the compound *vs* acarbose ($n = 3$, ANOVA, Tukey).
